# Focal DEPDC5 loss without disruption to cerebral cortical neuron migration recapitulates *DEPDC5*-related focal epilepsy

**DOI:** 10.1172/jci.insight.181544

**Published:** 2025-09-25

**Authors:** Karenna J. Groff, Yini Liang, Christopher Morici, Jinita B. Modasia, Leena Mehendale, Nishtha Gupta, Angelica D’Amore, Yongho Choe, Mustafa Q. Hameed, Alexander Rotenberg, Mustafa Sahin, Christopher J. Yuskaitis

**Affiliations:** 1 F.M. Kirby Neurobiology Center, Department of Neurology, Boston Children’s Hospital, Harvard Medical School, Boston, Massachusetts, USA.; 2 Rosamund Stone Zander Translational Neuroscience Center, and; 3 Division of Epilepsy and Clinical Neurophysiology and Epilepsy Genetics Program, Boston Children’s Hospital, Boston, Massachusetts, USA.

**Keywords:** Genetics, Neuroscience, Epilepsy, Genetic diseases, Seizures

## Abstract

Focal cortical dysplasia (FCD) is a major cause of refractory epilepsy and is associated with pathogenic variants in mTOR pathway genes, including *DEPDC5*, the most common cause of familial focal epilepsy. The mechanisms of epileptogenesis associated with FCD and hyperactive mTOR signaling remain unclear in *DEPDC5*-related epilepsy. To test whether DEPDC5 loss leading to seizures requires in utero cortical developmental defects or whether postnatal neuronal dysfunction of mTORC1 is sufficient to drive seizures, we developed a postnatal focal cortical *Depdc5*-knockout mouse model. Postnatal day 0–1 *Depdc5*-floxed mice received unilateral motor cortex injections of either AAV-Cre-GFP or control AAV-GFP. The AAV-Cre-GFP–injected hemisphere had decreased DEPDC5 levels with hyperactivation of mTOR that increased with age compared with both the contralateral hemisphere and the AAV-GFP–injected mice. Cortical lamination was not disrupted by postnatal DEPDC5 loss. Pathologic hallmarks of FCDs were identified in the *Depdc5*-knockout hemisphere, including increased SMI-311 neurofilament staining, hypomyelination, astrogliosis, and microglial activation. Mice with postnatal cortical DEPDC5 loss exhibited lower seizure thresholds, increased focal seizures, and increased rates of seizure-induced death compared with control mice. This study demonstrates that postnatal DEPDC5 loss and subsequent mTOR hyperactivation without disruption of cortical migration is sufficient to cause epilepsy.

## Abstract 2

<strong>Loss of *DEPDC5* gene function after early brain development can still lead to epilepsy in mice providing critical insight into the developmental windows that lead to epilepsy and implications for genetic rescue.</strong>

## Graphical abstract

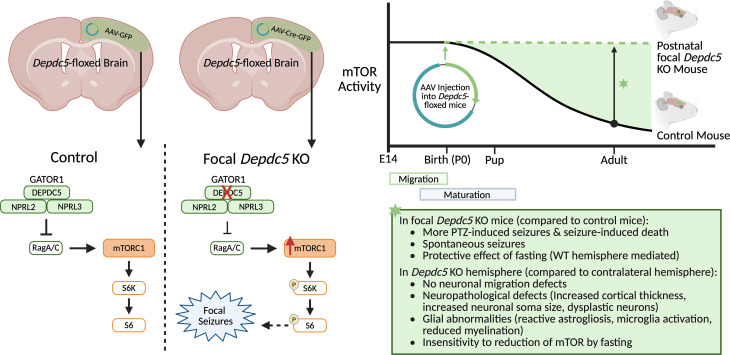

## Introduction

Focal cortical dysplasias (FCDs) are malformations of the cerebral cortex that represent the most common etiology of medically refractory epilepsy and neocortical epilepsy in children (1, 2). FCD type II (FCD II) is the most common subtype, characterized by disruption of the laminar cortical structure, dysmorphic neurons (type IIa), and balloon cells (type IIb) (1). Pathogenic variants in the mechanistic target of rapamycin (mTOR) pathway genes (e.g., *DEPDC5*, *NPRL2*, *NPRL3*, *MTOR*, *RHEB*, and *AKT3*) account for up to 60% of FCD II cases (3). Hyperactivation of mTOR complex 1 (mTORC1) is often identified in FCDs (4). In utero models leading to neuronal mTOR hyperactivity influence the severity of later epilepsy and associated neuropathology of FCDs (5). However, it is unclear whether the pathological hallmarks of FCD and seizures require in utero disruption of neuronal development or can be recapitulated by their effects on postnatal function.

DEP domain–containing protein 5 (DEPDC5) is part of the GATOR1 subcomplex that inhibits mTORC1 activity (6). mTORC1 signaling regulates cell growth and cell proliferation, including neuronal growth. DEPDC5 inactivation leads to mTORC1 hyperactivity both in vivo and in vitro (6, 7). This mTOR hyperactivity accounts for many of the histological features of FCD, especially cytomegaly (1).

*DEPDC5*-related epilepsy is the most common genetic cause of familial focal epilepsy (8–10). FCD II is commonly identified in patients with pathogenic loss-of-function variants in *DEPDC5* (8, 11–13). We previously generated a neuron-specific conditional *Depdc5*-knockout mouse model (*Depdc5^c/c^*-*Syn1*^Cre^) by Cre recombination under the synapsin 1 promoter (14). *Depdc5^c/c^*-*Syn1*^Cre^ neuron-specific-knockout mice were able to survive to adulthood and recapitulated the epileptic and neuropathological effects that are common features of mTORC1 hyperactivity, including decreased seizure threshold, macrocephaly, and increased neuron size (14). Similar findings were reported in an *Emx*-Cre *Depdc5*-knockout mouse model (15) and in *Depdc5*-heterozygous mice with brain-specific knockout (16). In utero electroporation focal *Depdc5*-knockout mouse models demonstrate neuronal migratory abnormalities in addition to increased mTORC1 and seizure activity (17–19).

All existing *Depdc5*-related epilepsy mouse models result in loss of DEPDC5 before or during neurogenesis. Most cortical neurogenesis in mice occurs between embryonic days 11 and 18 (20, 21). In the *Emx*-Cre model, the *Emx1* promoter drives Cre recombinase expression beginning on embryonic day 10 (17, 18). In the *Syn*-Cre model, the *Syn1* promoter drives Cre-recombinase expression beginning on embryonic day 12–13 (14, 15, 17). In the in utero electroporation models, embryos were electroporated on embryonic day 14.5 (19). Each of these models report either defective neuronal migration, ectopic neurons, or both (20). Therefore, it remains unclear whether the seizure phenotypes reported are a result of abnormal migration or postnatal neuronal mTORC1 signaling defects.

We hypothesized that DEPDC5 loss leading to sustained hyperactivation of mTORC1 signaling alone without neuronal migration defects would be sufficient to cause neuropathological hallmarks of FCD, seizures, and seizure-induced death in a focal epilepsy mouse model. To test this hypothesis, we created a focal *Depdc5*-knockout mouse model wherein *Depdc5* knockout occurred after the completion of neurogenesis. We based our model generation on a prior focal PTEN-knockout mouse model, in which mice with a *loxP*-flanked exon 5 of the PTEN gene received adeno-associated viral (AAV)-Cre-GFP injections into the sensorimotor cortex, leading to postnatal focal PTEN deletion (22). We adapted this methodology to create a mouse model of focal *DEPDC5-*related epilepsy without neuronal migration defects via unilateral stereotactic injection of AAV-Cre-GFP into the motor cerebral cortex of postnatal day zero (P0) or P1 *Depdc5*-floxed (*Depdc5^c/c^* or *Depdc5^c/–^*) mouse pups.

Here, we report a postnatal, focal *Depdc5*-knockout mouse model, which despite displaying no cerebral cortical neuronal migration defects, has a lower seizure threshold, increased seizure-induced death, and unilateral evidence of mTOR hyperactivation with FCD II–like neuropathological changes and associated glial abnormalities. The current study is the first to our knowledge to demonstrate that postnatal loss of DEPDC5 and subsequent hyperactivation of mTORC1 signaling is sufficient to recapitulate the *DEPDC5-*related epilepsy phenotypes of FCD II pathology and seizures.

## Results

### mTOR activity over time.

**Figure 1:**
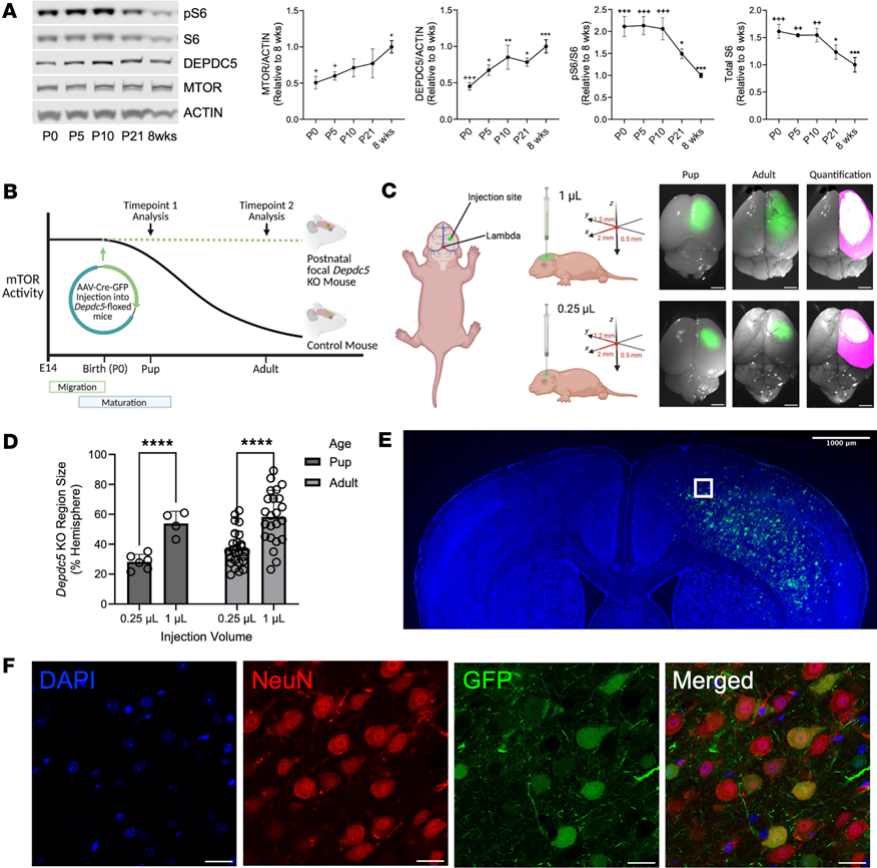
mTOR activity in mouse brain over time and generation of a postnatal focal brain knockout mouse model. (**A**) Immunoblots and quantitative analysis from cortical brain lysates of P0, P5, P10, P21, and 8-week-old control mice (*n* = 3) demonstrate a decrease in mTORC1 activity over time. Graph of mean ± SD. *P < 0.05, **P < 0.01, ***P < 0.001 compared with P0; ^+^*P* < 0.05, ^++^*P* < 0.01, ^+++^*P* < 0.001 compared with 8 weeks, all by 1-way ANOVA with Tukey’s post hoc analysis. (**B**) Overview of postnatal, focal *Depdc5*-knockout method and time points of analysis. (**C**) Schematic of small (0.25 μL) or large (1 μL) injection volume of 2 × 10^9^ genome copies of AAV-Cre-GFP at P0–P1. Representative images or pup and adult mice, with pink representing the entire hemisphere and white the GFP area used for quantification. (**D**) Injection volume correlates with AAV-Cre-GFP viral uptake. Max entropy thresholding quantification from pup and adult mice injected with AAV-Cre-GFP. Graph shows mean ± SD (circles represent individual animals). ****P < 0.0001 by 2-way ANOVA with Holm-Šidák correction for multiple comparisons. (**E**) Representative coronal section of AAV-Cre-GFP–injected *Depdc5^c/–^* adult mice. DAPI, blue; GFP, green. Scale bar: 1000 μm. (**F**) NeuN staining of coronal brain sections from *Depdc5*-knockout region of AAV-Cre-GFP–injected *Depdc5^c/–^* adult mice. DAPI, blue; NeuN, red; GFP, green. Scale bars: 25 μm. Representative images taken from *n* ≥ 4 mice per group.

We first interrogated the mTORC1 pathway in the cerebral cortex of wild-type mice over postnatal development. In cortical brain lysates, there was an increase in total protein mTOR and DEPDC5 levels from P0 to 8-week-old mice (Figure 1A). Downstream of mTORC1, there was a significant reduction in phosphorylation of Ser240/Ser244 in S6 (p-S6), a marker of mTORC1 activity, throughout development even when adjusting for a decrease in total S6 levels (Figure 1A). These data demonstrate a postnatal reduction in mTORC1 activity in the developing brain (16, 23, 24) as well as an expected inverse correlation between mTORC1 activity and DEPDC5, a known negative regulator of mTORC1 activity.

### Generation of postnatal focal Depdc5-knockout mice.

We then sought to test whether early postnatal DEPDC5 loss leads to persistent mTORC1 hyperactivity and increased seizure susceptibility in the absence of cortical neuron migration defects. Towards this end, we injected P0–P1 *Depdc5-*floxed (*Depdc5^c/c^* or *Depdc5^c/–^*) pups with 2 × 10^9^ genome copies of either AAV-Cre-GFP or AAV-GFP into the motor cortex of the right hemisphere and analyzed at pup (P8–P10) and adult (P50–P80) time points (Figure 1, B and C). Larger injection volumes of viral solution with the same viral titer resulted in a significantly larger area of viral uptake that was stable as animals aged (Figure 1, C and D). Examination of coronal brain sections from AAV-Cre-GFP–injected *Depdc5-*floxed mice showed colocalization of GFP, the viral transduction reporter, with NeuN throughout the cortical layers, confirming successful neuronal transduction (Figure 1, E and F).

**Figure 2:**
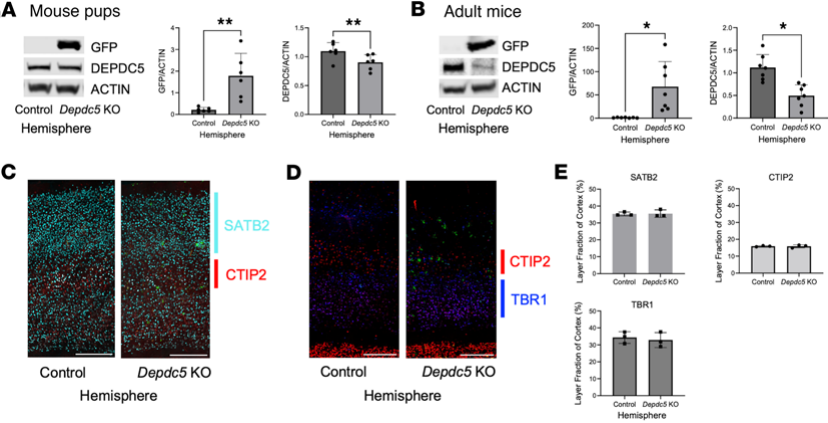
Confirmation of DEPDC5 loss without cortical migration defects in postnatal *Depdc5* focal knockout model. (**A** and **B**) Immunoblots and quantitative analysis of cortical brain lysates from the control and AAV-Cre-GFP–injected hemisphere of *Depdc5^c/c^* pups (**A**, *n* ≥ 5) and adult mice (**B**, *n* = 7) demonstrate successful *Depdc5* knockout in AAV-Cre-GFP–injected hemisphere (referred to as *Depdc5* KO). Graph of mean ± SD. Expression of levels were normalized to β-actin. *P < 0.05, **P < 0.01 by paired Student’s *t* test. (**C** and **D**) Representative image of anatomically matched cortical sections (*n* = 7) from *Depdc5*-knockout and control hemisphere. Scale bars: 250 μm. (**E**) Cortical layer thickness measurements at 4 paired sites in at least 3 sections per brain (*n* = 3) from each cortical layer marker (SATB2, CTIP2, and TBR1) in 1 μL–injected focal knockout *Depdc5^c/–^* mice (paired Student’s *t* test). Graph of mean ± SD.

We verified DEPDC5 loss at the AAV-Cre-GFP injection sites in *Depdc5-*floxed mice. In both pups and adult mice, we saw an increase in GFP levels in the *Depdc5* cortical knockout region compared with the contralateral hemisphere, indicating transduction by AAV-Cre-GFP in the knockout region (Figure 2, A and B). At both time points, we observed a statistically significant decrease in DEPDC5 levels in the AAV-Cre-GFP–injected region by Western blotting (Figure 2, A and B), which will henceforth be referred to as the *Depdc5*-knockout region.

We examined the cortical laminar structure of postnatal focal *Depdc5-*knockout mice. Immunofluorescent staining and quantification of the layer fraction of cortical layer VI (TBR1) (25), cortical layer V (CTIP2) (26, 27), and cortical layers II–IV (SATB2) (28) were not significantly different in the *Depdc5*-knockout hemisphere compared to the contralateral hemisphere (Figure 2, C–E). Similarly, no differences in neuronal migration were observed between hemispheres when comparing layer fraction of cortex in different genotypes (*Depdc5^c/c^* versus *Depdc5^c/–^*) or different injection volumes (0.25 μL versus 1 μL) (Supplemental Figure 1; supplemental material available online with this article; https://doi.org/10.1172/jci.insight.181544DS1).

### Age-dependent mTOR activity and neuropathologic changes in focal Depdc5-knockout cortex.

**Figure 3:**
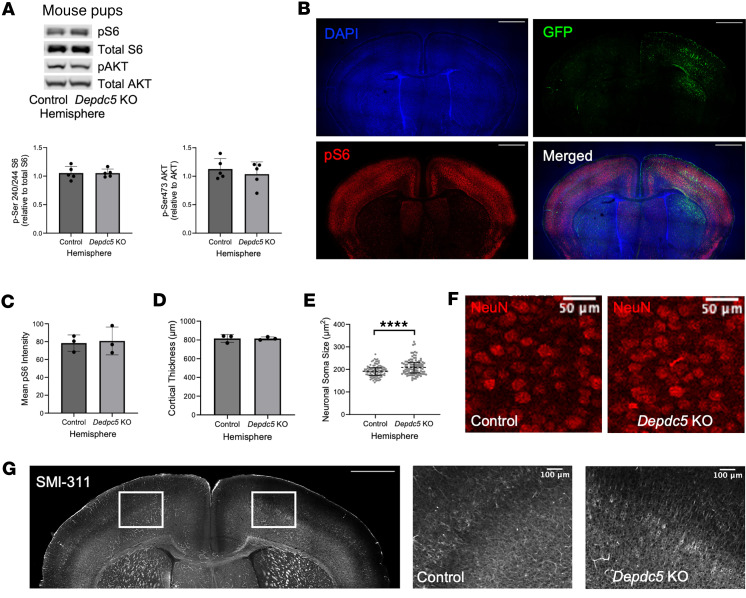
Cortical injection of AAV-Cre-GFP shows minimal downstream effects in mouse pups. (**A**) Immunoblots and quantitative analysis of cortical brain lysates from the control and AAV-Cre-GFP–injected hemisphere of *Depdc5^c/c^* pups (*n* = 5) demonstrate no statistically significant downstream effects in mTOR signaling. Expression of levels were normalized to total levels of S6 and AKT for p-S6 (S240/S244) and p-AKT (S473), respectively. (**B**) Representative immunohistochemistry of p-S6 (scale bars: 1000 μm) and (**C**) quantification of mean p-S6 intensity of mTORC1 activity in the *Depdc5*-knockout region versus the control hemisphere of AAV-Cre-GFP–injected *Depdc5^c/–^* mice (*n* = 3). (**D**) Cortical thickness measured at 4 paired sites in at least 3 sections per AAV-Cre-GFP–injected brain (*n* = 3) comparing the *Depdc5*-knockout and control hemispheres. (**E** and **F**) Quantified neuronal soma area (**E**) and representative NeuN (red) immunohistochemistry (**F**) from AAV-Cre-GFP–injected *Depdc5^c/–^* pups (*n* = 3). Scale bars: 50 μm. Quantification by ImageJ from 100 layer V NeuN^+^ cortical neurons from matched locations of M1 motor cortex per hemisphere shows larger neuron soma size in the *Depdc5*-knockout region compared with the control hemisphere. Two-way ANOVA interaction: *F*(99, 400) = 0.9736, *P* = 0.5541; individual neurons: *F*(99,400) = 0.8637, *P* = 0.8095; hemisphere: *F*(1,400) = 17.51, *P* < 0.0001. (**G**) Early dysplastic neurons within *Depdc5*-knockout hemisphere are evident by SMI-311 (white) staining (*n* = 3). Control hemisphere (middle) with minimal linear SMI-311 staining. *Depdc5*-knockout hemisphere (right) with stronger SMI-311 staining. Scale bars: 1000 μm (left) and 100 μm (middle, right). ****P < 0.0001 by paired Student’s *t* test. Dots represent individual mice (**A**, **C**, and **D**) or neurons (**E**). All graphs of mean ± SD.

We assessed for evidence of mTORC1 hyperactivation 1 to 10 weeks following postnatal, focal *Depdc5* knockout. Evidence of mTOR hyperactivation in response to *Depdc5* knockout was minimal in pups. Immunoblotting revealed no significant change in p-S6 or p-AKT levels in the *Depdc5*-knockout region compared to the control hemisphere (Figure 3A). No significant difference in p-S6 intensity in the *Depdc5-*knockout region was observed (Figure 3, B and C).

The gross morphology of the postnatal, focal *Depdc5-*knockout cortical region in pups was normal; we found no difference in cortical thickness between hemispheres (Figure 3D). However, NeuN-immunoreactive (NeuN^+^) neurons in the *Depdc5*-knockout region were significantly larger than those in the control hemisphere (*P* < 0.0001) (Figure 3, E and F). Dysplastic, enlarged neurons are a characteristic feature of mTOR hyperactivity and have been observed in humans with *DEPDC5* variants (14, 29, 30). Since dysplastic neurons typically exhibit abnormal cytoplasmic accumulation of argyrophilic fibrils and increased expression of neurofilament proteins (14, 29, 31), we used expression of non-phosphorylated neurofilament (SMI-311) to detect dysplastic neurons in our postnatal, focal *Depdc5*-knockout mice. Coronal brain sections from pups exhibited consistent SMI-311 staining across hemispheres limited to sparse layer III and V neurons (Figure 3G). Neuronal projections were aligned toward the cortical surface, as expected (32). In the *Depdc5*-knockout hemisphere in pups, some cytomegalic neurons with thicker, constrained dendritic arbors were apparent; however, the difference between hemispheres was subtle (Figure 3G).

**Figure 4:**
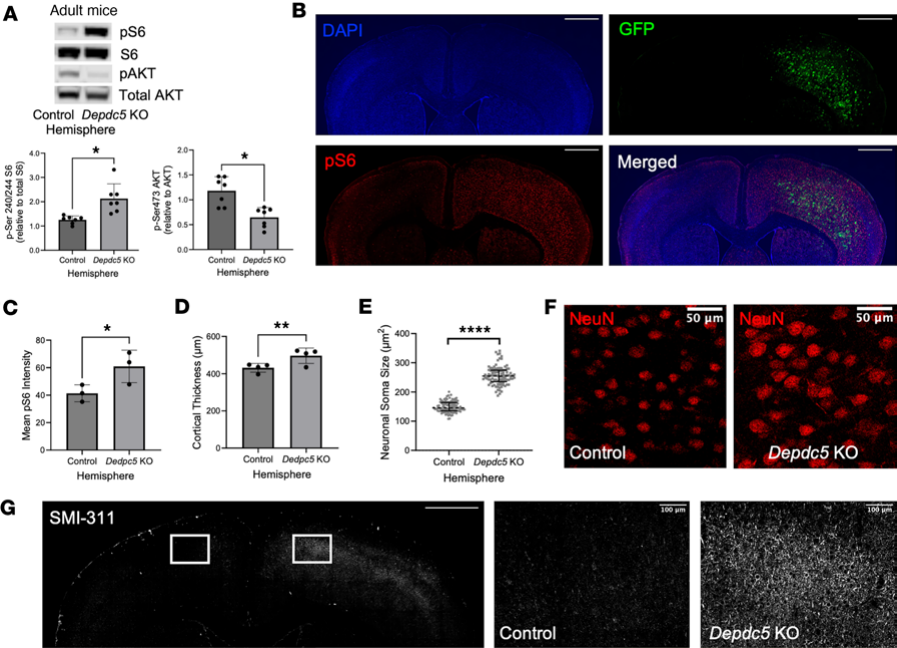
Cortical injection of AAV-Cre-GFP shows clear neuropathological effects in the *Depdc5*-knockout hemisphere of adult mice. (**A**) Immunoblots and quantitative analysis of cortical brain lysates from the control and AAV-Cre-GFP–injected hemisphere of *Depdc5^c/c^* mice (*n* = 7). Expression of levels were normalized to total levels of S6 and AKT for p-S6 (S240/S244) and p-AKT (S473), respectively (**B**) Representative immunohistochemistry of p-S6 (scale bars: 1000 μm) and (**C**) quantification of mean p-S6 intensity of mTORC1 activity in the *Depdc5*-knockout region versus the control hemisphere of AAV-Cre-GFP–injected *Depdc5^c/–^* mice (*n* = 3). For consistency, the same brain section is shown in Figure 1E and Figure 4B. (**D**) Cortical thickness measured at 4 paired sites in at least 3 sections per AAV-Cre-GFP–injected brain (*n* = 3) comparing the *Depdc5*-knockout and control hemispheres. (**E** and **F**) Neuron soma size is larger in the *Depdc5*-knockout region than control hemisphere. Quantified neuronal soma area (**E**) and representative NeuN (red) immunohistochemistry (**F**) from AAV-Cre-GFP–injected *Depdc5^c/–^* pups (*n* = 3). Scale bars: 50 μm. Quantification by ImageJ from 100 layer V NeuN^+^ cortical neurons from matched locations of M1 motor cortex per hemisphere (*n* = 3 mice). Two-way ANOVA interaction: *F*(99, 400) = 0.6531, *P* = 0.9943; individual neurons: *F*(99,400) = 1.016, *P* = 0.4487; hemisphere: *F*(1,400) = 722.0, *P* < 0.0001. (**G**) Dysplastic neurons within *Depdc5*-knockout hemisphere are evident by SMI-311 (white) staining (*n* = 3). Control hemisphere (middle) with minimal linear SMI-311 staining. *Depdc5*-knockout hemisphere (right) with strong, disorganized SMI-311 staining. Scale bars: 1000 μm (left) and 100 μm (middle, right). *P < 0.05, **P < 0.01, ****P < 0.0001 by paired Student’s *t* test. Dots represent individual mice (**A**, **C**, and **D**) or neurons (**E**). All graphs of mean ± SD.

In adult mice, we observed common features of mTORC1 hyperactivity within the *Depdc5*-knockout region. Cortical lysates from the *Depdc5*-knockout region had a significant increase in p-S6 (*P* < 0.05) compared with control lysates after normalizing to total S6 levels (Figure 4A). AKT phosphorylation was also significantly decreased (*P* < 0.05) (Figure 4A). Immunohistochemistry findings confirmed the intense p-S6 staining in the *Depdc5*-knockout regions of the brain, but not in the control hemisphere (Figure 4, B and C). The intensity of p-S6 staining was the same between hemispheres in adult AAV-GFP–injected mice (Supplemental Figure 2A).

In adult mice, the cortical thickness in the *Depdc5*-knockout region was significantly greater than in the control hemisphere (*P* < 0.01) (Figure 4D). Additionally, in adult mice the neuronal soma size in the *Depdc5*-knockout region was significantly larger than those in the control hemisphere (*P* < 0.0001) (Figure 4, E and F). In adult mice, there was a stark difference in SMI-311 staining between the *Dedpc5*-knockout and control hemispheres (Figure 4G). The control hemisphere showed limited SMI-311 staining. In contrast, *Depdc5*-knockout tissue showed strong SMI-311 staining throughout the knockout region. Many SMI-311^+^ layer V neurons displayed thicker dendritic arbors and disorganized projections with an abnormal orientation. No hemispheric differences in SMI-311 staining were observed in adult AAV-GFP–injected mice (Supplemental Figure 2A).

Taken together, these results suggest that focal, cortical reduction of DEPDC5 results in neuropathological defects that are common features of mTORC1 hyperactivity despite minimal disruption to cortical development. Furthermore, the effects result from prolonged mTORC1 hyperactivity and will be subtle, if present at all, in pups.

### Glial pathology in the Depdc5-knockout cortical hemisphere.

**Figure 5:**
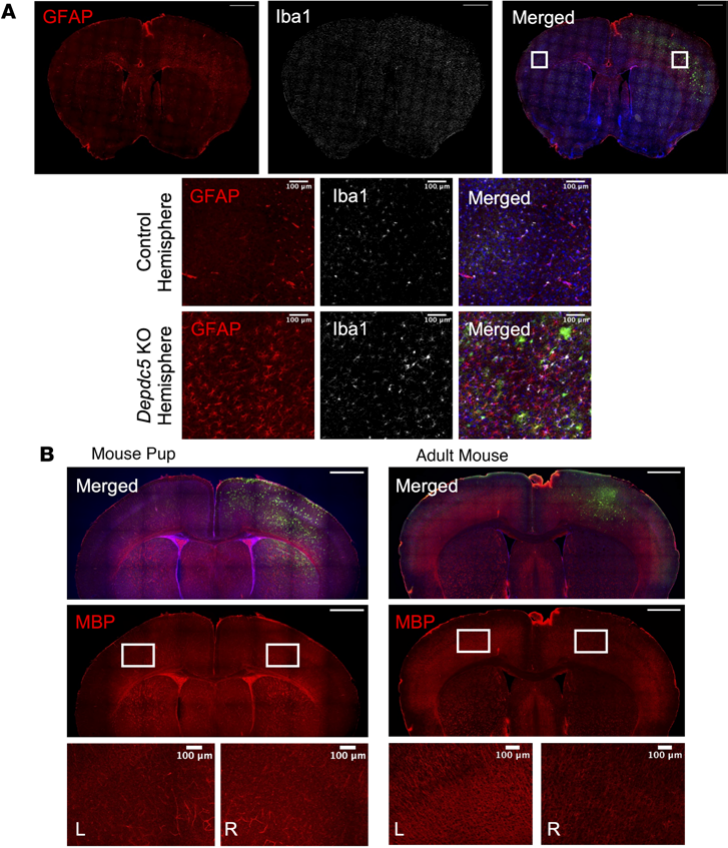
Evidence of glial abnormalities in postnatal focal *Depdc5*-knockout mice. (**A**) Top: Immunohistochemistry shows evidence of reactive astrogliosis (GFAP) and microglial activation (Iba1) in the *Depdc5*-knockout region of the right cortex compared with the control hemisphere of adult *Depdc5^c/–^* mice (P50–P80) previously injected with AAV-Cre-GFP on P0 (*n* = 3). Scale bars: 1000 μm. Bottom: Higher magnification shows reactive astrogliosis (GFAP) and activated microglia (Iba1) with abnormally large cell bodies in the *Depdc5*-knockout hemisphere. Scale bars: 100 μm. GFAP, red; Iba1, white; merged with DAPI and GFP. (**B**) Left: No evidence of hypomyelination in *Depdc5*-knockout region of AAV-Cre-GFP–injected *Depdc5^c/–^* pups compared to control hemisphere (*n* = 3). Right: The cerebral cortex of AAV-Cre-GFP–injected *Depdc5^c/–^* adult mice (*n* = 3) shows a reduction in MBP expression compared with the control hemisphere, indicating hypomyelination. MBP, red; merged with DAPI and GFP. Scale bars: 1000 μm (top) and 100 μm (bottom).

We investigated the potential role of glia in the neurological phenotypes of mTORC1 hyperactivation by first evaluating astrogliosis, as generally reflected by increased glial-fibrillary acidic protein (GFAP) staining. In postnatal, focal *Depdc5*-knockout mice, increased GFAP staining and evidence of astrogliosis were present in the *Depdc5*-knockout hemisphere and absent in the control hemisphere of adult mice (Figure 5A). We also considered changes in microglial activation, which has been linked to the pathogenesis of the onset of epilepsy (33). We stained coronal brain sections for Iba1, a marker of microglia activation, and noted a widespread increase in microglia activation in the cortex of the *Depdc5*-knockout hemisphere as opposed to the control hemisphere (Figure 5A). Some microglia appeared to have distinctly larger cell bodies (Figure 5A), another characteristic of microglia activation (34). No enlarged cell bodies or hemispheric differences in astrogliosis or microglia activation were observed in the AAV-GFP–injected adult mice (Supplemental Figure 2C). Lastly, we examined myelination in our focal *Depdc5*-knockout mice via immunohistochemical staining for myelin basic protein (MBP). While there was no discernable difference in MBP staining in pups, by adulthood reduced MBP expression was seen throughout the cortex of the *Depdc5*-knockout hemisphere (Figure 5B). MBP expression was also examined in AAV-GFP–injected adult mice, and no hemispheric differences were observed (Supplemental Figure 2B). These findings provide evidence for the dysfunction of glial cells, including astrocytes, microglia, and oligodendrocytes, in response to loss of DEPDC5.

### Focal Depdc5-knockout mice have decreased PTZ-induced seizure threshold, increased incidence of seizure-induced death, and abnormal EEG.

**Figure 6:**
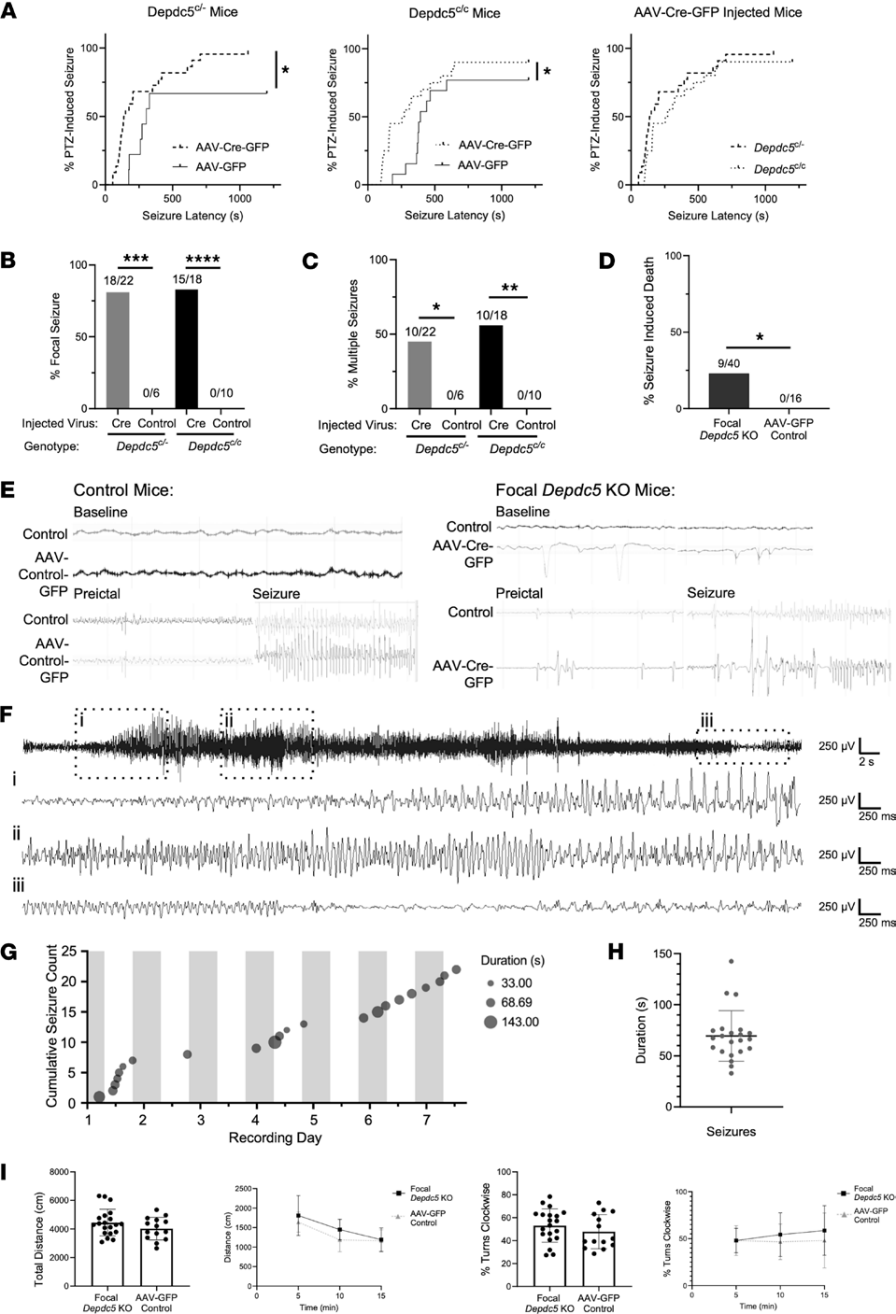
Focal *Depdc5* loss in mice results in decreased seizure threshold, focal seizures, and seizure-induced death. (**A**) After PTZ (65 mg/kg i.p. injection), there was a significant reduction in seizure susceptibility between focal *Depdc5-*knockout (AAV-Cre-GFP–injected) and control mice (AAV-GFP–injected) for both *Depdc5*-floxed genotypes (*Depdc5^c/–^* and *Depdc5^c/c^*). There was no difference in seizure susceptibility between *Depdc5^c/–^* mice and *Depdc5^c/c^* mice (*Depdc5^c/c^*: AAV-Cre-GFP *n* = 22, AAV-GFP *n* = 9; *Depdc5^c/c^*: AAV-Cre-GFP *n* = 20, AAV-GFP *n* = 13). Gehan-Breslow-Wilcoxon test. (**B**–**D**) Of the mice that seized after PTZ, a significantly greater proportion of focal *Depdc5*-knockout mice exhibit focal seizures contralateral to the injected hemisphere (**B**), multiple seizures during the 20-minute monitoring period (**C**), or seizure-induced death (**D**) compared with control mice. Fisher’s exact test. (**E**) Representative 2-channel EEG traces of PTZ-induced seizures at baseline, preictal, and ictal times, with consistent asymmetries between the 2 hemispheres (control vs. AAV-injected) in focal *Depdc5*-knockout mice as compared with control mice. (**F**) Single-channel EEG trace of the (i) beginning, (ii) evolution, and (iii) resolution (with postictal slowing) of a typical spontaneous convulsive seizure in a focal *Depdc5*-knockout mouse. (**G**) Cumulative spontaneous convulsive seizure burden in a focal *Depdc5*-knockout mouse across 1 week of continuous EEG recording (circles represent individual seizures with diameters indicating duration; gray bars indicate daily dark phase). (**H**) Average spontaneous convulsive seizure duration in a focal *Depdc5*-knockout mouse (whiskers represent SD). (**I**) Focal *Depdc5* loss does not result in hyperactivity in mice. Total distance traveled and percentage clockwise turns is similar between focal *Depdc5*-knockout mice (*n* = 20) and control mice (*n* = 14) throughout the 15-minute open-field paradigm (Student’s *t* test). Graphs show mean ± SD. *P < 0.05; **P < 0.01; ***P < 0.001; ****P < 0.0001.

We used an acute pentylenetetrazol-induced (PTZ-induced) seizure paradigm to compare seizure threshold in focal *Depdc5*-knockout mice with control AAV-GFP–injected mice (35). Compared with controls, focal *Depdc5* knockout in both *Depdc5^c/–^* mice and *Depdc5^c/c^* mice significantly decreased seizure threshold. There was no significant difference in seizure latency between the 2 genotypes, suggesting that they could be combined for future threshold analyses (Figure 6A). PTZ injection in *Depdc5^c/–^* mice led to seizures in 100% of those previously injected with AAV-Cre-GFP, while only 67% of AAV-GFP–injected mice had seizures (*P* < 0.05) (Figure 6A). Focal *Depdc5* knockout in *Depdc5^c/c^* mice increased the incidence of PTZ-induced seizures from 77% to 90% (*P* < 0.05) (Figure 6A).

Given the focal nature of *Depdc5* loss in our mice, we assessed the prevalence of generalized PTZ-induced seizures that initially showed a focal phenotype. These seizures were characterized by unilateral, repetitive, stereotyped jerking on one side of the body, and unidirectional rolling behavior during a seizure. This characteristic rolling behavior followed by unilateral weakness in the postictal phase was observed multiple times and always contralateral to the injected hemisphere. We observed a focal phenotype in 82% of *Depdc5^c/–^* mice, 83% of *Depdc5^c/c^* mice, and 0% of control mice (Figure 6B).

The PTZ-induced seizure phenotype in focal *Depdc5*-knockout mice was severe. During the 20-minute monitoring period, 45% of *Depdc5^c/–^* mice and 56% of *Depdc5^c/c^* mice experienced multiple generalized tonic-clonic seizures (GTCs), compared with none of the control mice (Figure 6C). Additionally, 22.5% of *Dedpc5*-knockout mice died following PTZ-induced seizures, while none of the control mice exhibited this phenotype (*P* < 0.05) (Figure 6D).

Electrographic evaluation of the focality of seizures was performed in a separate cohort, with electrodes implanted into both the control and *Depdc5*-knockout hemisphere. A representative EEG trace shows asymmetry between hemispheres in the focal *Depdc5* knockout, both during the baseline recording and during the preictal and ictal phase of the PTZ-induced seizure, with no such asymmetry observed in the control mice (Figure 6E).

During normal handling circumstances, we observed spontaneous seizures in 2 focal *Depdc5*-knockout mice: a P79 *Depdc5^c/–^* mouse injected with 0.25 μL of AAV-Cre-GFP and a P69 *Depdc5^c/c^* mouse injected with 0.5 μL of AAV-Cre-GFP. The seizure in the *Depdc5^c/–^* mouse was noted to be focal, with a distinct unilateral rolling phenotype. We therefore performed longitudinal, week-long video electroencephalography (EEG) recordings in 3 focal *Depdc5*-knockout mice to evaluate the incidence of epilepsy. Our results indicate that focal knockout triggers an epileptogenic process that results in spontaneous convulsive seizures (Figure 6F; representative EEG traces) in a time-dependent manner. None of the recorded mice seized at the first time point (~P90), while 1 of 3 exhibited multiple spontaneous convulsive seizures at the second recording time point (~P120; Figure 6G). The mouse experienced an average of 3.1 seizures/day, with a mean seizure duration of 69.5 seconds (Figure 6H). As with PTZ-induced seizures, spontaneous convulsive seizures were characterized by initial unilateral, repetitive, stereotyped jerking and rolling behavior (Supplemental Video 1).

### Focal Depdc5 loss did not result in hyperactivity or early mortality in mice.

DEPDC5 knockdown has been shown to cause mTOR dependent hyperactivity in both zebrafish and mice (36, 37). Interestingly, adult postnatal, focal *Depdc5*-knockout mice exhibited no significant increase in locomotor activity compared to control mice in the open-field assay (Figure 6I). We assessed the percentage of clockwise turns to provide a readout of potential unilateral hyperactivity resulting from focal *Depdc5* knockout. Despite a trend toward an increase in the percentage of clockwise turns in focal *Depdc5*-knockout mice compared with controls, this difference was not statistically significant (Figure 6I). Therefore, we conclude that postnatal, focal *Depdc5*-knockout mice exhibit no evidence of hyperactivity.

We previously reported that full-brain knockout *Depdc5^c/c^*-*Syn1*^Cre^ mice exhibit decreased survival, with a median lifespan of 123 days (37). To evaluate survival, we assessed whether mice died spontaneously by 200 days of age. We monitored 1 μL AAV-Cre-GFP–injected *Depdc5^c/–^* mice (*n* = 5) and *Depdc5^c/c^* mice (*n* = 7), as well as AAV-GFP–injected control mice (*n* = 10). All mice survived up to 200 days, indicating no evidence of early mortality in postnatal, focal *Depdc5*-knockout mice.

### Acute fasting had a subtle protective effect from seizures in focal Depdc5-knockout mice.

**Figure 7:**
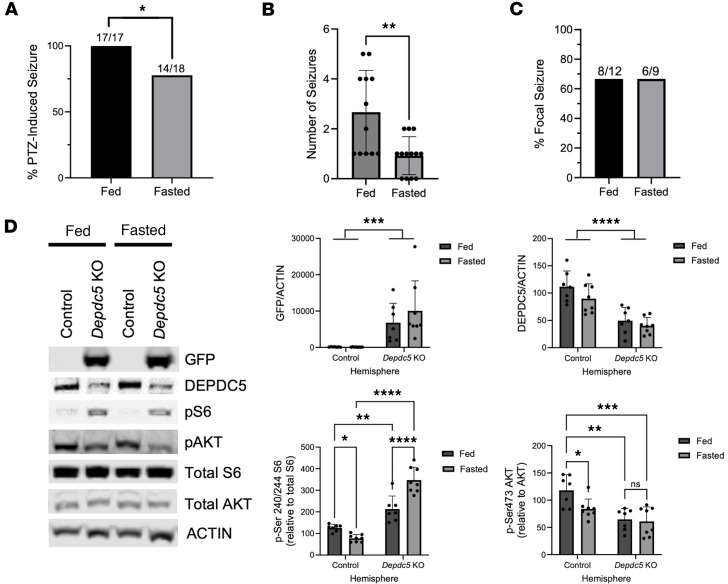
Fasting is protective of PTZ-induced seizures in focal *Depdc5*-knockout mice. (**A**) After PTZ (65 mg/kg i.p. injection), there was a statistically significant decrease in seizure susceptibility between fed and fasted focal *Depdc5^c/c^* mice. *P < 0.01 by χ^2^ test. (**B**) After PTZ, the number of seizures observed during the 20-minute monitoring period significantly decreased in focal knockout *Depdc5^c/c^* mice with fasting. Graph of mean ± SD. **P < 0.01 by Student’s *t* test. (**C**) Of the mice that experience seizures after PTZ, there was no change in the frequency of focal seizures in fed mice as compared to fasted mice. Fisher’s exact test. (**D**) Brain mTORC1 activity in the *Depdc5*-knockout hemisphere was insensitive to the protective effects of fasting. Immunoblots from cortical brain lysates and relative quantifications demonstrate a significant increase in GFP levels and decrease in DEPDC5 levels in the *Depdc5*-knockout hemisphere compared with the control hemisphere, and no change with 24-hour fasting. Immunoblots and relative quantifications demonstrate a reduction in p-S6 (S240/S244) and p-AKT (S473) after 24-hour fasting in the control hemisphere, while no such reduction is observed in the *Depdc5*-knockout hemisphere. There is an increase in p-S6 levels and decrease in p-AKT levels in the *Depdc5*-knockout hemisphere compared with the control hemisphere. Graphs of mean ± SD. *P < 0.05; **P < 0.01; ***P < 0.001; ****P < 0.0001 by 2-way ANOVA with Holm-Šidák correction for multiple comparisons.

Acute fasting has been shown to protect from seizures and reduce mTOR activity in the brain in a DEPDC5-dependent fashion (38). We tested whether 24-hour fasting protects adult *Depdc5^c/c^* mice from seizures in an acute PTZ-induced seizure paradigm. Fasting for 24 hours significantly reduced the percentage of mice exhibiting PTZ-induced seizures from 100% to 77.8% (Figure 7A). Fasting also significantly reduced the number of seizures observed within the 20-minute monitoring period from 2.7 in the fed state to 0.9 in the fasted state (Figure 7B). There was no effect on seizure latency or duration (Supplemental Figure 3, A and B). Fasting did not affect the focality of seizures in *Depdc5^c/c^* mice, with both fed and fasted groups having focal seizures 66.7% of the time (Figure 7C). Despite a trend toward protection against seizure-induced death (17.6% of mice that had seizures dying in the fed state versus 7.1% dying in the fasted state), this difference was not statistically significant (Supplemental Figure 3C).

Next, we tested whether mTOR activity was altered in the cerebral cortex after fasting in the control versus *Depdc5*-knockout regions of the brain. By Western blot analysis of cortical lysates, fasting significantly reduced mTORC1 (p-S6 staining) and mTORC2 (p-AKT staining) activity in the control hemisphere of focal *Depdc5*-knockout mice (Figure 7D). No such decrease was observed with p-S6 or p-AKT in the *Depdc5*-knockout region of the brain (Figure 7D), suggesting that reduction in mTOR activity following fasting is mediated by DEPDC5, as previously demonstrated (38). Fasting had no effect on GFP or DEPDC5 levels in either the control or *Depdc5*-knockout hemisphere (Figure 7D). In the *Depdc5*-knockout region compared with the control hemisphere, we observed a significant increase in GFP, decrease in DEPDC5 protein, increase in p-S6, and decrease in p-AKT (Figure 7D), as we previously demonstrated in Figure 2B and Figure 4A, further confirming effective AAV transduction.

## Discussion

The current study is the first to our knowledge to test whether DEPDC5 loss and GATOR complex disruption leads to epileptogenesis in the absence of neuronal migration deficits. This study demonstrates that postnatal neuronal dysfunction of mTORC1 signaling due to DEPDC5 loss is sufficient to recapitulate the FCD II neuropathology and focal seizures, even in the absence of in utero cortical neuron migration defects. Specifically, we noted the presence of dysmorphic neurons and glial abnormalities in the *Depdc5*-knockout region, consistent with findings from patient samples (30, 39, 40). We also found an increase in focal seizures and seizure-induced death in postnatal focal *Depdc5*-knockout mice compared with control mice. Our preclinical mouse model can support testing of gene-based therapies for patients with *DEPDC5-*related epilepsy and FCD.

As a master regulator of intracellular signaling, mTORC1 regulates many functions during brain development, including neuronal proliferation, differentiation, migration, and maturation (41). Reduced mTORC1 activity has been shown to reduce neural progenitor cell populations (42). Hyperactivation of mTORC1 results in aberrant migration of daughter cells (41). DEPDC5, as a part of the GATOR1 complex, modulates mTORC1 activity through amino acid–sensing mechanisms distinct from tuberous sclerosis complex (TSC) (38). The distinct role of DEPDC5 in early neuronal development has only recently been studied. All prior animal models of *Depdc5*-related epilepsy knocked out *Depdc5* either before or during neurogenesis and demonstrated aberrant neuronal migration in addition to sustained postnatal dysfunction in mTORC1 signaling (34). Our approach to generate a focal *Depdc5*-knockout model without defective neuronal migration was aimed to avoid the embryonic timeframe of neuronal migration by knocking out *Depdc5* postnatally. Normal neuronal migration was observed, as measured by unchanged cortical layer thickness and expression of layer-specific markers between *Depdc5*-knockout and control hemispheres of the brain. Furthermore, we showed that mTORC1 activity is highly dynamic over time, with a rapid decrease postnatally around P10. By knocking out *Depdc5* prior to P10, we recapitulated the same sustained postnatal dysfunction in mTORC1 signaling as seen in previous models (35).

In young mice (≤P10), a postnatal reduction in DEPDC5 had minimal physiological consequences. We confirmed neuronal transduction with the AAV-Cre-GFP vector by increased GFP and a modest decrease in DEPDC5 expression in the injected region of the brain. There were no changes in p-S6 signaling, p-AKT signaling, or cortical thickness, although increased neuronal soma size and dysplastic neurons were observed in these mice. A small reduction in DEPDC5 levels at this early age might explain the subtle phenotype. It is also possible that high levels of mTOR activity in neonates represent a physiological ceiling that limits the effect of DEPDC5 loss. If so, the natural age-related decrease in mTOR activity unmasks the sustained hyperactive mTOR phenotype in the regions with reduced DEPDC5 levels.

DEPDC5 reduction was more pronounced when the mice reached adulthood. We demonstrate that postnatal DEPDC5 loss results in mTORC1 hyperactivation in adult mice in the *Depdc5*-knockout region. This finding is consistent with previous studies showing increased p-S6 levels with DEPDC5 reduction both in vivo (7, 14, 18) and in vitro (6). An expected reduction in mTORC2 activation was observed via decreased p-AKT levels in the *Depdc5*-knockout region compared with the control hemisphere (37). This reduction in p-AKT levels in *Depdc5*-knockout mice is likely mediated by feedback signaling from hyperactive mTORC1 (38). While our results corroborate prior data from other *DEPDC5* models, this is the first model to our knowledge to demonstrate that postnatal DEPDC5 reduction is sufficient to drive much of the overall pathology and phenotype.

By adulthood, postnatal focal *Depdc5*-knockout mice exhibited many neuropathological defects consistent with mTORopathies. Humans with heterozygous loss-of-function *DEPDC5* variants exhibit epilepsy with or without developmental cortical malformations such as FCD. FCD II is one of the most common neuropathological findings in tissues resected from these patients and is characterized by laminar disorganization (17, 43). Other common neuropathological hallmarks of FCD II include increased cortical thickness, increased neuronal cell body size, and dysmorphic neurons (17, 43, 44). Interestingly, despite having normal cortical architecture, focal knockout mice recapitulated these neuropathological hallmarks in the *Depdc5*-knockout region of the cortex. We found evidence of increased cortical thickness and neuronal soma size in the *Depdc5*-knockout hemisphere compared with the control hemisphere. Full-brain *Depdc5*-knockout mice with migration defects also show increased cortical thickness and neuronal enlargement in response to DEPDC5 reduction (41). We observed dysplastic cortical neurons in the *Depdc5*-knockout region consistent with previous findings in full-brain *Depdc5*-knockout models (42). The presence of this phenotype in the postnatal mouse model provides evidence that dysplastic cortical neurons can develop subsequent to neuronal migration. Taken together, our results indicate that mTORC1 hyperactivity alone is sufficient to cause neuropathological effects characteristic of DEPDC5 loss and FCD II. These effects are not apparent in young postnatal *Depdc5*-knockout mice because they are driven by persistently elevated mTORC1 activity.

A lower seizure threshold in mice with postnatal focal loss of DEPDC5 was observed in a PTZ-induced seizure paradigm. The main reason to challenge mice with PTZ to determine whether they had a lower seizure threshold was based on the infrequent spontaneous seizures in the full-brain *Depdc5*-knockout mouse model (42). After PTZ injection, only focal *Depdc5*-knockout mice and not control mice exhibited a focal seizure behavior phenotype. The localized region of focal seizures in knockout mice was confirmed by EEG recordings showing asymmetry between the injected and uninjected hemispheres, which was not observed in control mice. To validate the results of the PTZ-induced seizures, we performed longitudinal EEG and video recording capturing spontaneous seizures and demonstrated that focal *Depdc5* knockout triggers an epileptogenic process that results in spontaneous, convulsive seizures. These results indicate that focal mTORC1 hyperactivation is sufficient to cause epileptic seizures, which are often focal-onset and may be followed by seizure-induced death.

Focal mTORC1 hyperactivation was not sufficient, however, to cause early mortality or behavioral hyperactivity in postnatal, focal *Depdc5*-knockout mice. One reason for the lack of an observed early mortality phenotype may be that we did not monitor focal knockout mice for a long enough period. We observed both spontaneous seizures and death following PTZ-induced seizures. Full-brain *Depdc5*-knockout mice showed spontaneous seizures, death following PTZ-induced seizures, and decreased survival due to seizure-induced death, with a median survival of 123 days (43). We monitored mice for 200 days and observed no death occurrences; however, death was observed in focal *Depdc5*-knockout mice beyond the 200-day monitoring period. Due to the less severe phenotype of focal *Depdc5*-knockout mice compared with full-brain knockout mice, this monitoring period was likely too short to observe early mortality. Lack of long-term survival monitoring is therefore a limitation of this study and warrants further investigation to adequately characterize spontaneous seizure-induced death in the focal *Depdc5*-knockout mice.

Neuropsychiatric comorbidities such as attention deficit hyperactivity disorder (ADHD) are common in patients with *DEPDC5-*related epilepsy (45). Both early mortality and hyperactivity have been shown in previous full-brain *Depdc5*-knockout models, supporting our finding that focal DEPDC5 loss results in a less severe seizure phenotype compared with generalized loss of DEPDC5 (44). Due to the postnatal nature of this focal *Depdc5*-knockout model, the absence of these phenotypes suggests that both early mortality and hyperactivity may be driven by either structural cortical migration defects or by other brain regions. ADHD has been associated with structural changes such as incomplete maturation of the middle and superior temporal gyrus and fronto-basal portions of both frontal lobes (46). The focal cortical *Depdc5* knockout generated here was primarily localized to the sensorimotor cortex of the frontal lobe. Our postnatal *Depdc5*-knockout model offers a useful tool to investigate the localization of various phenotypes within the brain, with the ability to adjust the coordinates of the AAV-Cre-GFP injection to knock out *Depdc5* in different regions of interest.

Increased risk of death following PTZ-induced seizures was observed in postnatal, focal *Depdc5*-knockout mice compared with controls. Sudden unexplained death in epilepsy (SUDEP) has been reported in patients with epilepsy who have pathogenic loss-of-function variants in *DEPDC5* (12, 13). These results support clinical evidence that loss of *DEPDC5* confers an increased risk of SUDEP. They also provide evidence that mTORC1 hyperactivity in the absence of any cortical migration abnormalities is sufficient to cause seizure-induced death in a PTZ-induced seizure model. The pathophysiology and molecular basis of SUDEP remains unknown; however, our findings suggest a potential cortical contribution to this phenotype. In the adult brain, serotonin neurons project to the majority of cortical areas. The focal *Depdc5* knockout generated here is localized primarily to the frontal lobe, which contains the highest density of serotonergic axons and serotonin receptors (47, 48). Serotonergic dysfunction has been implicated in the pathogenesis of SUDEP (49, 50). Neuronal DEPDC5 loss has been shown to reduce serotonin levels and provoke seizure-induced death in rodent models (47). Seizure-induced death was absent in control mice and only observed in the focal *Depdc5*-knockout mice after PTZ. While PTZ-induced seizure models have been used to study SUDEP, there are many limitations to the use of a PTZ-induced model (51). Further studies would require validation in other *Depdc5* mouse models with spontaneous seizure-induced death.

Our data provide further insight into the effects of fasting on mTOR and seizure propensity. Acute fasting has a protective effect from seizures by DEPDC5-mediated amino acid–sensing mechanisms (49). Full-brain DEPDC5 loss renders mice insensitive to this protective effect of fasting from seizures (51). We observed fasting-induced reduction in mTORC1 and mTORC2 activity in the non-injected control hemisphere of focal *Depdc5*-knockout mice. In contrast, the *Depdc5*-knockout region in the injected hemisphere was insensitive to reduction in mTORC1 activity from fasting, as measured by p-S6 levels. Nonetheless, 24-hour fasting had a seizure protective effect in focal knockout mice. It is possible that the seizure-protective reduction in mTOR activity facilitated by wild-type neurons was sufficient to protect knockout mice from seizures despite insensitivity to fasting in the focal knockout region. This model provides insight into the effects of fasting with partial DEPDC5 loss. Patients with focal *DEPDC5-*related epilepsy may benefit from the protective effects of fasting through DEPDC5-mediated amino acid sensing in wild-type neurons.

The seizure phenotypes observed in this focal knockout model may be a direct result of persistent hyperactivation of neuronal mTOR; however, glial changes may contribute. Previous studies have supported glial dysfunction in the pathophysiology of mTOR-related epilepsy in both mouse models and clinical studies of TSC (14, 52). Here, we show that focal *Depdc5* loss results in astrogliosis and microglial activation, with no evidence of astrogliosis in the unaffected, contralateral hemisphere. Astrogliosis and microglial activation were absent in AAV-GFP–injected mice, suggesting that these neuroinflammatory changes do not represent procedure-related injury. These results are consistent with full-brain knockout models of both *Depdc5* and *Tsc1* with astrogliosis and microglia activation that are rescued by mTOR inhibitors (34, 52, 53). Additionally, both cell-autonomous effects of glial cells and their interactions with neurons and other cells have been shown to play a role in the pathophysiology of mTORopathies (52). Future studies with glia- and neuron-specific promoters are needed to fully characterize the cell-autonomous and non-cell-autonomous mechanisms underlying the neuroinflammation and epilepsy phenotype identified in *Depdc5*-knockout models.

In this study, reduced cortical myelination was noted in the hemisphere with DEPDC5 loss. We only observe hypomyelination in adult *Depdc5* focal knockout mice following persistent mTOR hyperactivation and not control AAV-GFP–injected mice. Hypomyelination and decreased oligodendrocyte number have been shown to occur in an mTOR-dependent manner in TSC1 mouse models (29, 54) and commonly found in FCD II (44). The absence of hypomyelination in pups with early DEPDC5 loss supports our hypothesis that hypomyelination is an effect of persistent mTOR hyperactivation. However, further work is needed to better understand the role of hypomyelination in *DEPDC5*-related epilepsy.

Given that DEPDC5 is a negative regulator, loss of heterozygosity may be necessary to develop FCD (55). A 2-hit mechanism has been proposed to explain this phenomenon, wherein a germline loss-of-function mutation is followed by a second somatic mutation in the brain, leading to cortical malformations and epilepsy (17, 55, 56). Importantly, the focal *Depdc5*-knockout mouse model presented here recapitulates the hypothesized 2-hit mechanism of DEPDC5-related familial focal epilepsy. *Depdc5^c/–^* mice have germline full-body heterozygous DEPDC5 loss. Cortical injection with AAV-Cre-GFP induces a second “somatic” mutation in the brain, which facilitates focal loss of heterozygosity. The current mouse model can aid in further elucidating the pathology of *Depdc5*-related epilepsy.

To our knowledge, we present the first rodent model of DEPDC5 loss without defects in cortical neuronal migration. Our findings provide compelling insight into the effects of persistent cortical mTORC1 hyperactivation. The current model targeted neuronal *Depdc5* knockout early in postnatal cortical development. P0 AAV2/8 injection predominantly results in viral uptake in neurons in the cortex (57). We avoided glial involvement by limiting injections to P0–P1, as glial transduction of AAV2/8 becomes predominant when the virus is administered after P2–P3 (57). The current postnatal *Depdc5*-knockout model leads to DEPDC5 loss after the completion of the cortical layer formation. However, neuronal maturation and especially interneuron development continues to occur postnatally (58). This study did not discern AAV transduction of excitatory and inhibitory interneurons. However, future postnatal injection studies could assess the relative roles of DEPDC5 in excitatory, inhibitory, and other neuronal subtypes by utilizing specific promoters. Future studies could also include regional specificity, targeting regions beyond the sensorimotor cerebral cortex. Nonetheless, the knockout model developed here offers a useful tool for interrogating the effects of DEPDC5 in focal brain regions.

In conclusion, our finding that postnatal focal loss of DEPDC5 is sufficient to cause seizures and neuropathological phenotypes associated with *DEPDC5-*related epilepsy demonstrates feasibility of gene-based therapy approaches to rescue DEPDC5 function in patients, and also provides an experimental platform for a range of future preclinical investigations.

## Methods

### Sex as a biological variable.

Our study examined male and female animals. Similar findings are reported for both sexes; however, the studies were insufficiently powered to address sex as a biological variable.

### Animals.

All mice were housed in a 12-hour light-dark cycle, climate-controlled room, with access to food and water ad libitum. Mouse experiments were performed in a mixed-strain background using equal numbers of male and female mice. Both *Depdc5^c/c^* and *Depdc5^c/–^* mice were used in this study. Conditional *Depdc5^c/c^* mice were generated as previously described (14). Heterozygous knockout *Depdc5^c/–^* mice, which have one conditional allele and one knockout allele, were generated using similarly published methods (16). *Depdc5^c/–^* males were bred with *Depdc5^c/c^* females to generate litters of both *Depdc5^c/–^* and *Depdc5^c/c^* mice for use in this study. During routine handling, animals were monitored for spontaneous convulsive seizures by visual inspection. Experiments were performed on both pup (P8–P10) and adult (P50–P80) mice.

When the cortex of the mouse brain is injected with AAV-Cre-GFP, conditional *Depdc5^c/c^* mice generate a focal *Depdc5*-knockout region in the brain, with all other cells in the body having wild-type *Depdc5*, whereas heterozygous knockout *Depdc5^c/–^* mice generate a focal *Depdc5-*knockout region in the brain with heterozygous loss of *Depdc5* in the rest of the body. In this way, heterozygous knockout *Depdc5^c/–^* mice better recapitulate the hypothesized 2-hit mechanism of *DEPDC5*-related familial focal epilepsy, wherein a germline loss-of-function mutation is followed by a second somatic mutation, leading to epilepsy (17).

### DNA analysis.

DNA was extracted from mouse toes and tails by standard procedures for genotyping. Genotyping at the *Depdc5* gene was performed using 2 separate primer pairs. First, a primer pair that allows for simultaneous analysis of wild-type, conditional, and knockout alleles (DEPDC5 Lox P1: 5′-TCCGCAAAGGTTAGGAGCTATG-3′, DEPDC5 Lox P2: 5′-CCCTCATGCCAGCTCAAACT-3′, and DEPDC5 Lox P3: 5′-TTG GTTCCCCTGAAACTGGG-3′), followed by agarose gel electrophoresis. An approximately 738 bp band was detected for the knockout allele, an approximately 430 bp band was detected for the conditional allele with the flanking *loxP* sites, and an approximately 367 bp band was detected for the wild-type allele (Supplemental Figure 4A). Second, we used a primer pair that allows for clear confirmation of the knockout allele (forward: 5′-AAGGCGCATAACGATACCAC-3′ and reverse: 5′-ACTGATGGCGAGCTCAGACC-3′), followed by gel electrophoresis. A 174 bp band was detected for the knockout allele (Supplemental Figure 4B).

### Neonatal focal cortical brain injections.

Prior to injection, each viral vector was diluted in sterile saline to a final titer of 2 × 10^9^ genome copies (GC). Virus was loaded into a 10 μL NanoFil Syringe (World Precision Instruments) with a 35-gauge beveled NanoFil Needle (World Precision Instruments).

P0–P1 pups were anesthetized by hypothermia, and then secured to a Stoelting Mouse & Neonatal Rat Adaptor using soft ear bars. Stereotaxic injection was performed using a Harvard Apparatus Syringe Pump and Model 940 Small Animal Stereotaxic Instrument with Digital Display Console (coordinates from lambda unless otherwise specified: AP 2 mm, ML 1.2 mm, DV 0.5 mm). Across different volumes of injection, the infusion flow rate was set such that the injection would proceed over a duration of 1 minute. Individual pups were identified by tail clipping. Following injection, pups were placed on a heating pad held at 37°C until fully awake, and then returned to their home cage. The syringe and needle were sterilized with enzymatic cleaners and dried with compressed dry air between uses.

### AAVs.

AAVs were sourced from the Boston Children’s Hospital Viral Core. The viral constructs used were AAV2/8-CMV-GFP-ires-cre (2.015 × 10^13^ GC/mL; referred to as AAV-Cre-GFP) and AAV2/8-CAG-GFP-WPRE (6.7 × 10^13^ GC/mL; referred to as AAV-GFP). These constructs encoded either a Cre recombinase–GFP fusion protein (AAV-Cre-GFP) or GFP (AAV-GFP) for a control. AAV2/8 capsids that were used as hybrid vectors have been reported to have a 5- to 100-fold higher efficiency for transgene delivery in the brain, as compared with AAV serotype 2 and other single serotypes (59, 60). Different promoters were used in each virus according to availability. CMV and CAG have previously been shown to confer comparable transgene expression levels and cellular transduction profiles (61).

Preliminary experiments with AAV-Control-GFP virus identified the optimal needle depth at 0.5 mm from the skull to accurately achieve cortical delivery of virus. We tested the volume of injection to examine for local spread throughout the cortex. Using the same viral load (total of 2 × 10^9^ GC), the area of AAV-Cre-GFP viral transduction increased with the injection volume (Figure 1D).

### Quantification of AAV-transduced cortical surface.

Quantification of the area of AAV transduction was performed using ImageJ software (NIH) and full-brain images acquired using a Zeiss SteREO Discovery.V20 stereomicroscope. The GFP channel of each image was converted to 8-bit and max entropy thresholding was used to define the lesion area (62). A mask of the lesion area was created, and its area was measured. A blinded observer used a freehand selection to outline and measure the area of the entire lesioned hemisphere (right) and the percentage of the hemisphere occupied by the lesion was calculated.

### Acute fasting paradigm.

Group-housed mice, 6–10 weeks old, were randomized to fed or fasted conditions. Mice were weighed followed by food removal in the early afternoon for 24 hours with ad libitum water access. Twenty-four hours after fasting, mice were weighed followed by experimentation for biochemistry or seizure susceptibility. For biochemistry, mice were sacrificed by guillotine with blood and brain rapidly collected. Mouse cerebral cortex was rapidly dissected and flash frozen. Blood glucose was measured immediately by Onetouch Ultra2 glucometer (LifeScan). Serum was collected by centrifugation at 1,000*g* for 10 minutes at 4°C and stored at –80°C.

### Acute PTZ seizure paradigm.

Pentylenetetrazol (PTZ; Sigma-Aldrich Co., P6500), a GABA_A_ receptor agonist, was dissolved fresh before each experiment in 1× PBS at 5 mg/mL. Group-housed mice, 6–10 weeks old from a stable mixed background line, were intraperitoneally (i.p.) injected with 65 mg/kg PTZ during the light phase of the light-dark cycle. Mice were monitored over a 20-minute period by an investigator blinded to genotype and injection type; our primary endpoint was GTCs, as previously described (35, 38). Latency of onset, duration, number of seizures, clonic jerks, death, and presence or absence of focal seizure were recorded. Focal seizures were characterized by unilateral, repetitive, stereotyped jerking on one side of the body or head, and unilateral rolling behavior during a seizure. After the 20-minute observation period, mice were sacrificed.

### EEG telemetry and analysis.

Adult mice underwent wireless telemetry transmitter (1-channel: PhysioTel ETA-F10; 2-channel: PhysioTel HD-X02; Data Sciences International) implantation as described previously (63, 64). All mice received analgesia for 72-hour postoperative pain relief (Ethiqa XR; 3.25 mg/kg subcutaneously). After 1 week of postoperative recovery, 1 week of epidural 1- or 2-channel continuous video-EEGs (sampling rate: 1 kHz) were recorded from each mouse (Ponemah Software v6.51, Data Sciences International). One-channel video-EEG data were screened for spontaneous GTCs using an automated seizure detection algorithm (NeuroScore 3.4.1, Data Sciences International). Automatically marked events were verified by visual review of real-time video and spectrogram (64), and GTC frequency and mean GTC duration were calculated. At the end of the recording period, animals were challenged with the *Acute PTZ seizure paradigm* described above.

### Open-field testing.

To check spontaneous locomotor activity, we used the open-field paradigm. All open-field testing was conducted in the Animal Behavior and Physiology Core at Boston Children’s Hospital Intellectual and Developmental Disabilities Research Center (IDDRC). Open-field testing was conducted on 35 mice between the ages of P62 and P65. Tests were performed blind to genotypes, and viral injection at the same point in the circadian cycle for all mice. Open-field experiments ran for 15 minutes, during which the mice freely moved in the circular open-field arena as previously described (36). Trials were recorded using Noldus EthoVision XT software. Both total distance and percentage of clockwise rotations were calculated to assess the effect of the unilateral *Depdc5* knockout. For rotation measurements, a minimum distance of 2 cm was used, with body axis rotations excluded from recording.

### Immunohistochemistry.

For immunohistochemistry, animals were euthanized and transcardially perfused with 4% paraformaldehyde. If immunohistochemistry followed a PTZ-induced seizure paradigm, then brains were instead drop fixed in 4% paraformaldehyde overnight at 4°C, after which they were stored in PBS. Full brain images were acquired using a Zeiss SteREO Discovery.V20 stereomicroscope prior to sectioning to assess for successful viral uptake and the presence of a GFP^+^ lesion. Coronal brain sections were sectioned with a vibratome at 50 μm using standard methods. Following 30 minutes of blocking (0.25% Triton X-100 and 5% normal goat serum in PBS), floating sections were immunolabeled overnight using the following primary antibodies in blocking buffer: anti–p-S6 (Ser240/Ser244) (Cell Signaling Technology, catalog 5364; 1:1000), anti-NeuN (MilliporeSigma, catalog ABN78; 1:1000), anti–SMI-311 (MilliporeSigma, catalog NE1017; 1:1000), anti-GFAP (Cell Signaling Technology, catalog 3670; 1:1000), anti-MBP (BioLegend, catalog 808401; 1:1000), anti-Iba1 (Fujifilm Wako, catalog 019-19741; 1:1000, anti-GFP (Abcam, catalog ab13970; 1:500), anti-CTIP2 (Abcam, catalog ab18465; 1:1000), anti-TBR1 (Abcam, catalog ab183032; 1:250), and anti-SATB2 (Abcam, catalog ab92446; 1:1000). Sections were washed 3 times, 5 minutes each, in PBS and the incubated for 90 minutes with the following secondary antibodies (diluted 1:1000 in blocking buffer, all from Thermo Fisher Scientific): goat anti-mouse Alexa Flour Plus 488 (catalog A32723), goat anti-mouse Alexa Flour Plus 555 (catalog A32727), goat anti-mouse Alexa Flour Plus 647 (catalog A32728), goat anti-rabbit Alexa Flour Plus 488 (catalog A32731), goat anti-rabbit Alexa Flour Plus 555 (catalog A32732), and goat anti-rabbit Alexa Flour Plus 647 (catalog A32733). Sections were then washed in PBS for 10 minutes and nuclei were stained with Hoechst 33342 (Thermo Fisher Scientific) for 5 minutes. Sections were then mounted with Fluoromount-G (SouthernBiotech). Images were acquired using a Leica TCS SP8 laser-scanning confocal microscope (10×, 20×, or 63× objective lens) or a Nikon 80i widefield microscope (2× or 20× objective lens). Images were configured and analyzed using ImageJ software. Blinded cortical thickness measurements were taken using the DAPI channel at a minimum of 4 locations per section, and a minimum of 3 anatomically matched sections per animal. Blinded cortical layer measurements were taken using the same methodology with the following layer markers delineating cortical layers: TBR1 expressed in layer VI and subplate (26), CTIP2 expressed in layer IV and layer VI (27), and SATB2 expressed in layers II–IV (29). Area measurement of layer V NeuN^+^ neurons were performed using ImageJ software by a blinded observer from at least 100 neurons in the M1 motor cortex per animal.

### Western blotting.

To evaluate the protein levels, protein extracts were prepared from dissected cortical tissue samples taken from the frontal cortex of *Depdc5^c/c^* and *Depdc5^c/–^* mice. For analysis of AAV-Cre-GFP–injected focal *Depdc5*-knockout mice, cortical tissue sections from the virally transduced region of the Cre-injected cortex were dissected using a Zeiss SteREO Discovery.V20 stereomicroscope under the 38 GFP filter block, allowing for visualization of the virally transduced region of the brain. Cortical tissue sections from size-matched contralateral and ipsilateral brain regions were also dissected via Zeiss SteREO Discovery.V20 stereomicroscope. Protein lysates were prepared on ice in RIPA buffer (Thermo Fisher Scientific) with protease and phosphatase inhibitors (Roche Diagnostics) followed by BCA protein assay (Thermo Fisher Scientific). Western blotting was performed using equal amounts of protein extract per lane (20–30 μg of protein) under reducing conditions in Bolt Bis-Tris 4%–12% SDS polyacrylamide precast gels (Thermo Fisher Scientific). Gels were transferred onto Immobilon-FL PVDF membranes (Millipore), blocked with Odyssey TBS blocking buffer (LI-COR) for 1 hour at room temperature, followed by primary antibody in Odyssey blocking buffer with 0.2% Tween 20 (Sigma-Aldrich) overnight at 4°C. Antibodies used for Western blotting were as follows: anti–β-actin (Cell Signaling Technology, catalog 3700; 1:5000), anti–p-S6 (Ser 240/Ser244) (Cell Signaling Technology, catalog 5364; 1:1000), anti-S6 (Santa Cruz Biotechnology, catalog sc-74459; 1:1000), anti–total Akt (Cell Signaling Technology, catalog 4691; 1:1000), anti–p-Akt Ser473 (Cell Signaling Technology, catalog 4060, 1:1000), anti-DEPDC5 (Sigma-Aldrich, catalog HPA055619; 1:1000), anti-GFP (Novus Biological, catalog NB600-308; 1:1000), and anti-mTOR (Cell Signaling Technology, catalog 2983S; 1:1000). After washing blots 3 times for 5 minutes each in Tris-buffered saline–Tween 20 (TBST), IRDye 800CW– or 680RD–conjugated secondary antibodies (1:10,000; LI-COR) in Odyssey blocking buffer with 0.2% Tween 20 and and 0.01% SDS were added for 1 hour at room temperature. To visualize bands, an Odyssey Fc Imager was used, and densitometry analysis was performed using Image Studio Software (LI-COR). Each band was normalized to the β-actin signal per sample. Levels of phosphorylated proteins were expressed as a ratio of phosphorylated to total protein level. All values were compared to the contralateral hemisphere as a control.

### Statistics.

Statistical analysis was performed using GraphPad Prism 9 software. The results are presented as mean ± SEM or SD. Comparisons between 2 independent groups were performed by unpaired, 2-tailed Student’s *t* test. Comparisons between hemispheres (control vs. *Depdc5* knockout) of mice were performed by 2-tailed, paired Student’s *t* test. Comparisons between multiple groups with 1 factor were performed by 1-way ANOVA with Tukey’s post hoc analysis. Comparisons between multiple groups with 2 factors were performed by 2-way ANOVA, sometimes with Holm-Šídák correction for multiple comparisons. Comparisons between seizure latency curves were performed using a Gehan-Breslow-Wilcoxon test. Contingency analysis was performed by either Fisher’s exact test or χ^2^ test. Alpha was set at 0.05 for significance and exact *P* values are reported for significant results.

### Study approval.

All mouse procedures were performed in accordance with the NIH *Guide for the Care and Use of Laboratory Animals* (National Academies Press, 2011), and the study was approved by the Institutional Animal Care and Use Committee and use of biological agents was regulated by the Institutional Biosafety Committee of Boston Children’s Hospital.

### Data availability.

Values for all data points in graphs are reported in the Supporting Data Values file.

## Author contributions

KJG, CM, and CJY conceived the study. KJG, YL, CM, JBM, LM, NG, ADA, YC, MQH, and CJY planned and performed experiments. KJG, YL, CM, JBM, ADA, MQH, and AR analyzed data. KJG and CJY wrote the manuscript. MS and CJY supervised all phases of the study. All authors read, revised, and approved the manuscript.

## Funding support

National Science Foundation Graduate Research Fellowship Program grant 2141064 (to KJG).

- NIH grant 1K08NS107637 (to CJY).
- NIH grant R01NS113591 (to MS).
- The Rosamund Stone Zander Chair (to MS).
- Hearst Foundation.
- Boston Children’s Hospital Translational Research Program.
- Boston Children’s Office of Faculty Development Career Development Fellowship.
- NIH grant P50HD105351 (to the BCH/Harvard Medical School Intellectual and Developmental Disabilities Research Center).
- The BCH Rosamund Stone Zander Translational Neuroscience Center.

## Supplementary Material

Supplemental data

Unedited blot and gel images

Supplemental video 1

Supporting data values

## Version 1 09/25/2025

In-Press Preview

## Version 2 11/10/2025

Electronic publication
