# Supplementary material for: Focal DEPDC5 loss without disruption to cerebral cortical neuron migration recapitulates DEPDC5-related focal epilepsy
Source: JCI Insight. 2025 Sep 25;10(21):e181544. doi: 10.1172/jci.insight.181544 (PMC12643507; doi:10.1172/jci.insight.181544)
Supplement: Supplemental data [file jciinsight-10-181544-s093.pdf]

Video 1. Spontaneous seizure in postnatal focal *Depdc5* knockout mouse. Seizure shows focal phenotype with unilateral repetitive jerking on one side of the body and unidirectional rolling/spinning.

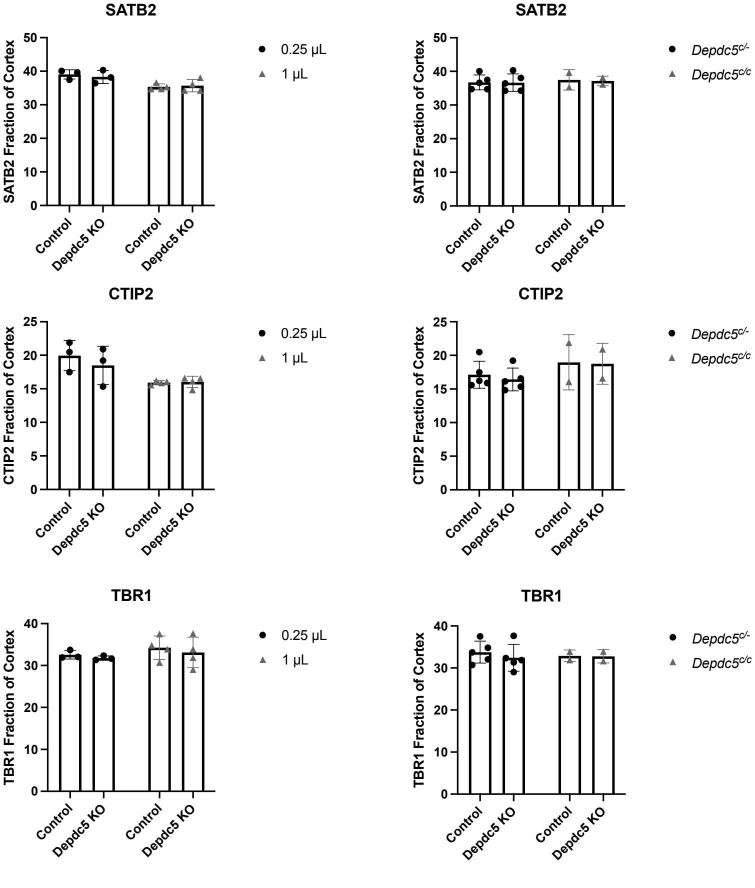
SATB2
SATB2 Fraction of Cortex
0 10 20 30 40 50
Control
Depdc5 KO
Control
Depdc5 KO
0.25 μL
1 μL
SATB2
SATB2 Fraction of Cortex
0 10 20 30 40 50
Control
Depdc5 KO
Control
Depdc5 KO
Depdc5c/-
Depdc5c/c
CTIP2
CTIP2 Fraction of Cortex
0 5 10 15 20 25
Control
Depdc5 KO
Control
Depdc5 KO
0.25 μL
1 μL
CTIP2
CTIP2 Fraction of Cortex
0 5 10 15 20 25
Control
Depdc5 KO
Control
Depdc5 KO
Depdc5c/-
Depdc5c/c
TBR1
TBR1 Fraction of Cortex
0 10 20 30 40
Control
Depdc5 KO
Control
Depdc5 KO
0.25 μL
1 μL
TBR1
TBR1 Fraction of Cortex
0 10 20 30 40
Control
Depdc5 KO
Control
Depdc5 KO
Depdc5c/-
Depdc5c/c

Supplemental Figure 1. Normal cortical architecture is maintained across genotype and injection volumes. Cortical layer thickness measurements at 4 paired sites in at least 3 sections per brain from each cortical layer marker (SATB2, CTIP2, and TBR1) at different injections volumes (0.25 μL or 1 μL) and in different genotypes (*Depdc5*$^{c/c}$ or *Depdc5*$^{c/-}$) (Two-way ANOVA).

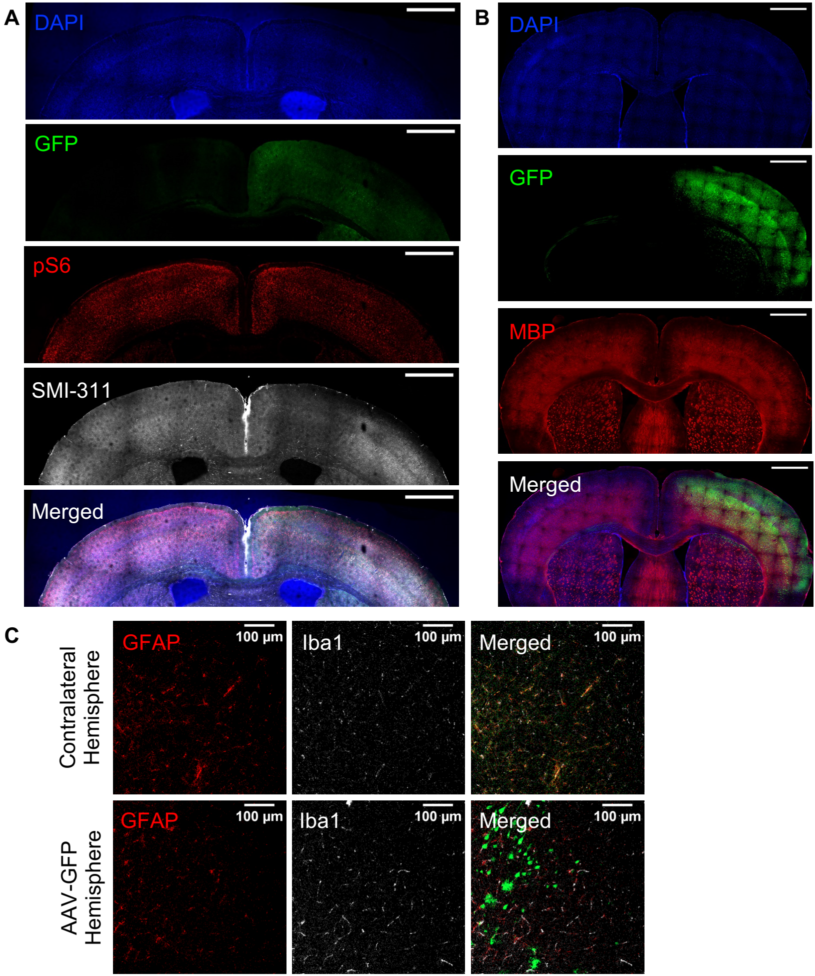
A
DAPI
GFP
pS6
SMI-311
Merged
B
DAPI
GFP
MBP
Merged
C
GFAP
100 μm
Iba1
100 μm
Merged
100 μm
Contralateral Hemisphere
GFAP
100 μm
Iba1
100 μm
Merged
100 μm
AAV-GFP Hemisphere

Supplemental Figure 2. No mTOR hyperactivation, neuropathological defects, or glial pathology are observed in control AAV-GFP injected mice. (A) Immunohistochemistry shows cortical uptake of AAV-GFP in the injected hemisphere of adult *Depdc5*$^{c/-}$ mice (n=5), but no change in the intensity of pS6 or SMI-311 staining between the two hemispheres, indicating no evidence of mTORC1 hyperactivity (pS6) or dysplastic neurons (SMI-311). DAPI, blue; GFP, green; pS6, red; SMI-311, white; Merged with DAPI, GFP, pS6, and SMI-311. Scale bar: 1000 μm. **(B)** Immunohistochemistry shows cortical uptake of AAV-GFP in the injected hemisphere of adult *Depdc5*$^{c/-}$ mice (n=3), but no change in the intensity of MBP staining between the two hemispheres, indicating no evidence of hypomyelination. DAPI, blue; GFP, green; MBP, red; Merged with DAPI, GFP, and MBP. Scale bar: 1000 μm. **(C)** Immunohistochemistry shows no difference in reactive astrogliosis (GFAP) or microglial activation (Iba1) between the AAV-GFP injected hemisphere and the contralateral hemisphere of adult *Depdc5*$^{c/-}$ mice (n=3). GFAP, red; Iba1, white; Merged with GFAP, Iba1, and GFP. Scale bar: 1000μm.

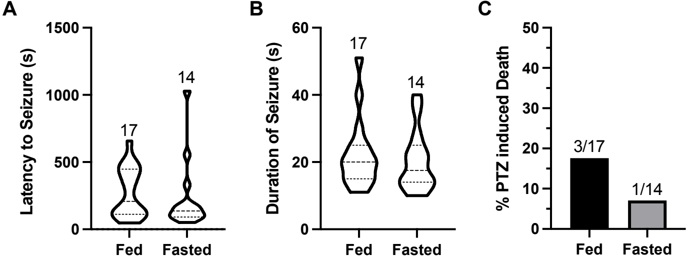
A
Latency to Seizure (s)
1500
1000
500
0
17
14
Fed
Fasted
B
Duration of Seizure (s)
60
40
20
0
17
14
Fed
Fasted
C
% PTZ induced Death
50
40
30
20
10
0
3/17
1/14
Fed
Fasted

Supplemental Figure 3. Fasting has no impact on seizure latency, seizure duration, or seizure-induced death in focal *Depdc5* knockout mice. (A) Seizure latency was not significantly different between fed and fasted focal *Depdc5*$^{c/c}$ knockout mice. (Dashed lines show median, dotted lines show IQR) (Student's t-test) **(B)** Seizure duration was not significantly different between fed and fasted focal *Depdc5*$^{c/c}$ knockout mice. (Dashed lines show median, dotted lines show IQR) (Student's t-test) **(C)** Seizure-induced death was not significantly different between fed and fasted focal *Depdc5*$^{c/c}$ knockout mice. (Fisher's exact test)

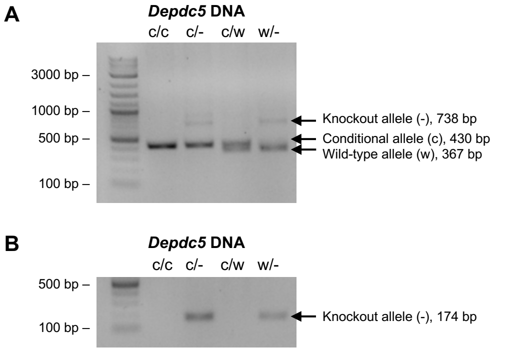
A
Depdc5 DNA
c/c c/- c/w w/-
3000 bp –
1000 bp –
500 bp –
100 bp –
Knockout allele (-), 738 bp
Conditional allele (c), 430 bp
Wild-type allele (w), 367 bp
B
Depdc5 DNA
c/c c/- c/w w/-
500 bp –
100 bp –
Knockout allele (-), 174 bp

Supplemental Figure 4. PCR product confirms the presence of knockout, conditional, and wild-type alleles. (A) PCR product delineates presence of knockout allele (-), conditional allele (c) or wild-type allele (w) in *Depdc5* DNA samples of various genotypes. **(B)** A secondary primer pair provides confirmation of the presence of a knockout allele (-) in *Depdc5*$^{c/-}$ and *Depdc5*$^{w/-}$ mice.
