# Supplementary material for: Focal DEPDC5 loss without disruption to cerebral cortical neuron migration recapitulates DEPDC5-related focal epilepsy
Source: JCI Insight. 2025 Sep 25;10(21):e181544. doi: 10.1172/jci.insight.181544 (PMC12643507; doi:10.1172/jci.insight.181544)
Supplement: Unedited blot and gel images [file jciinsight-10-181544-s094.pdf]

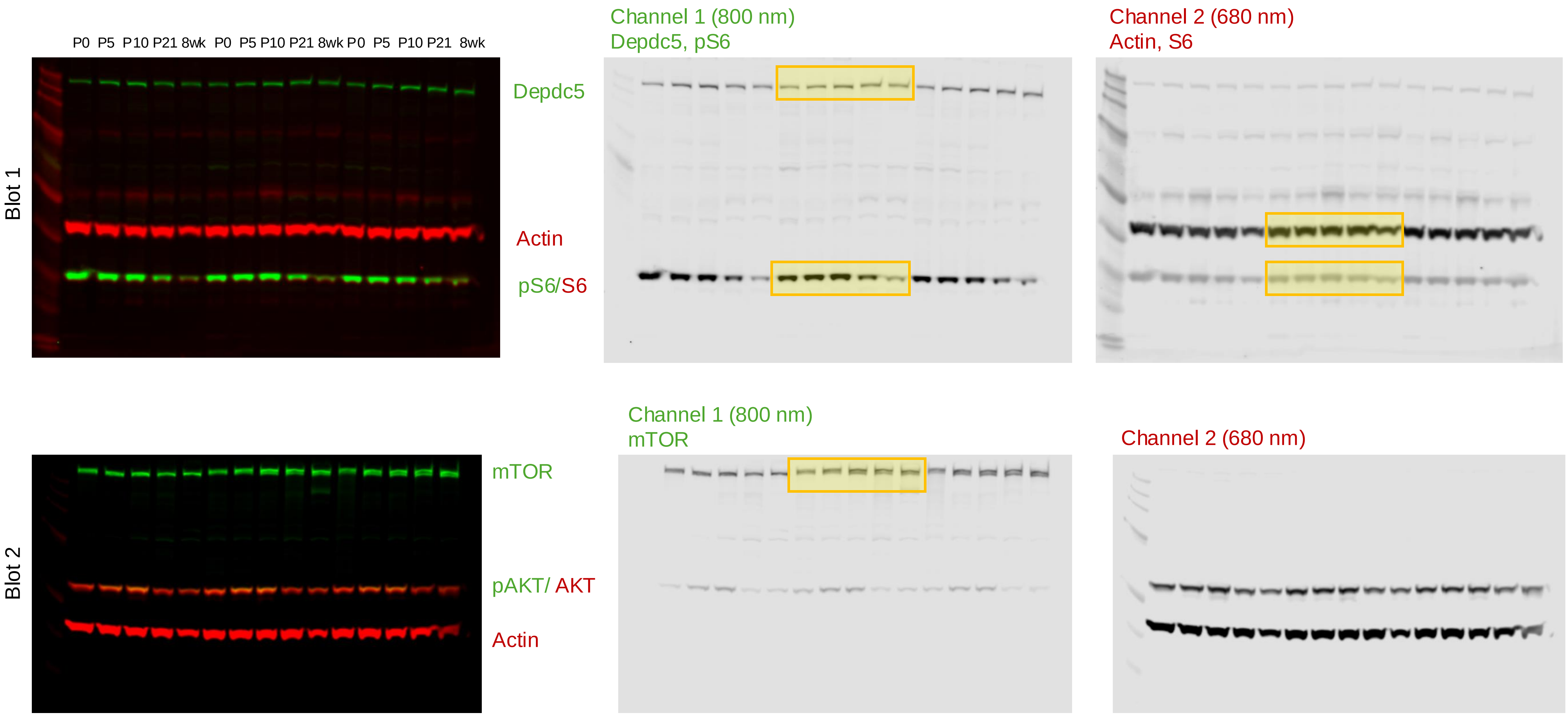

Full unedited gel for Figure 1A

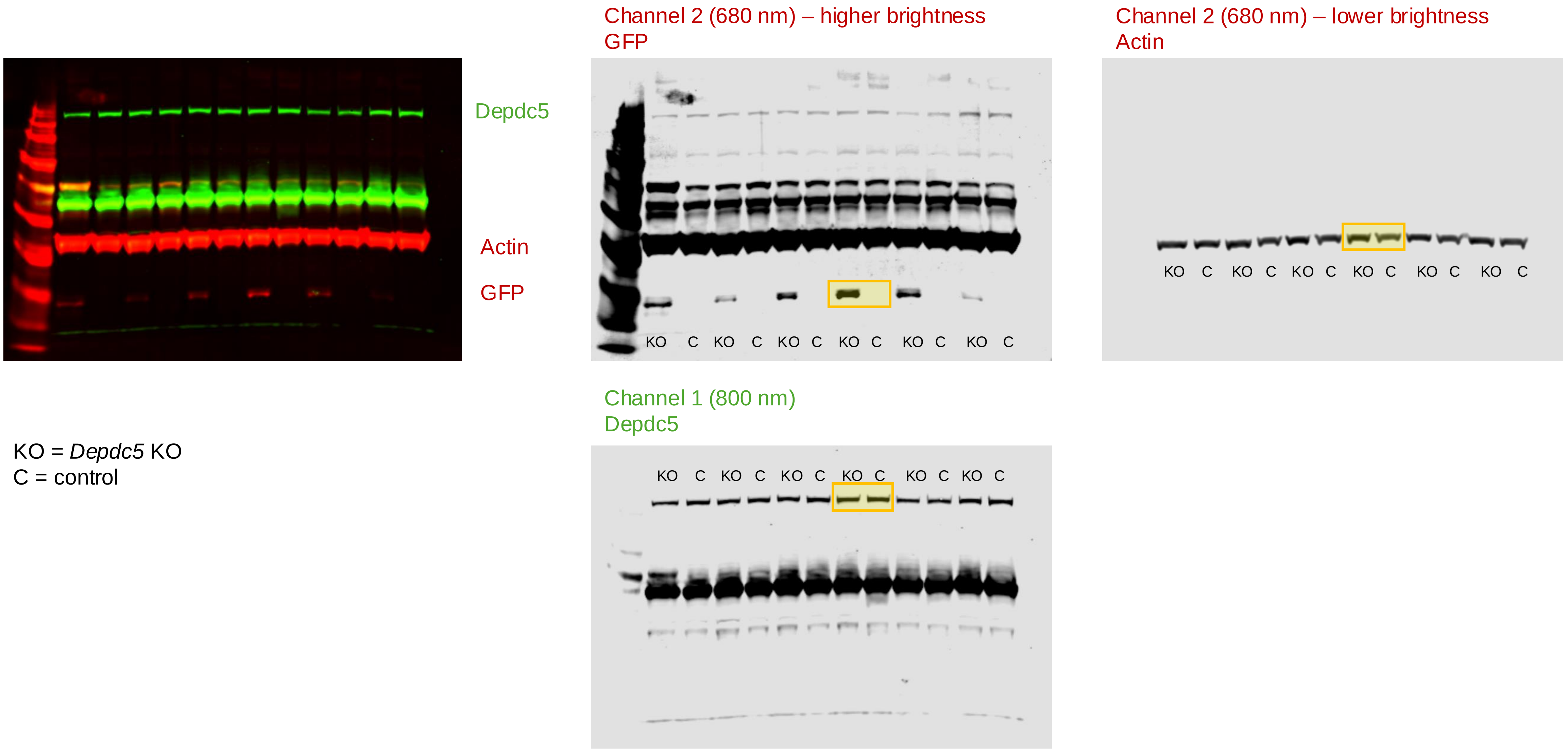

Full unedited gel for Figure 2A

GFP

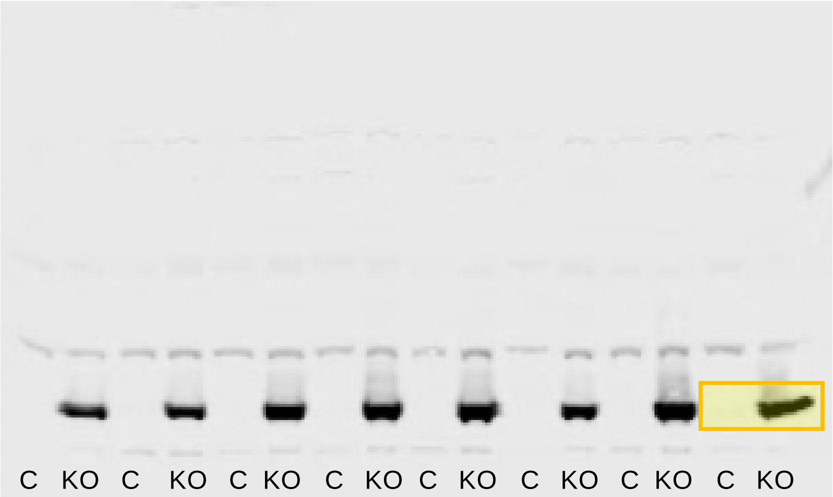

Depdc5

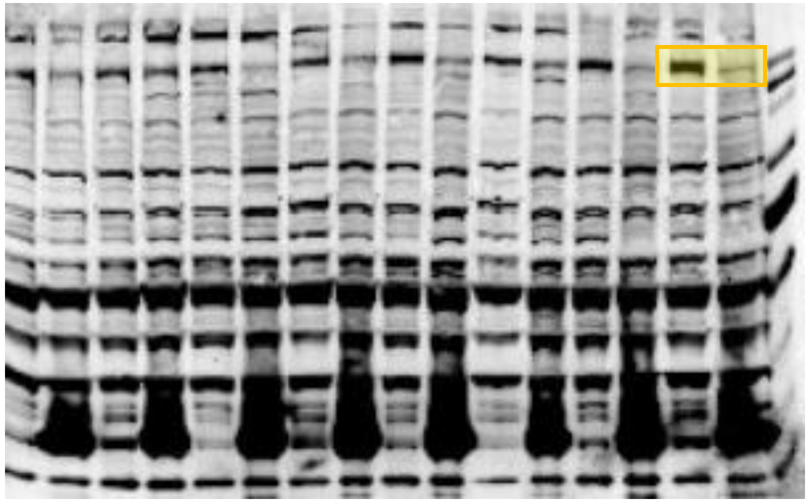

C KO C KO C KO C KO C KO C KO C KO C KO

Actin

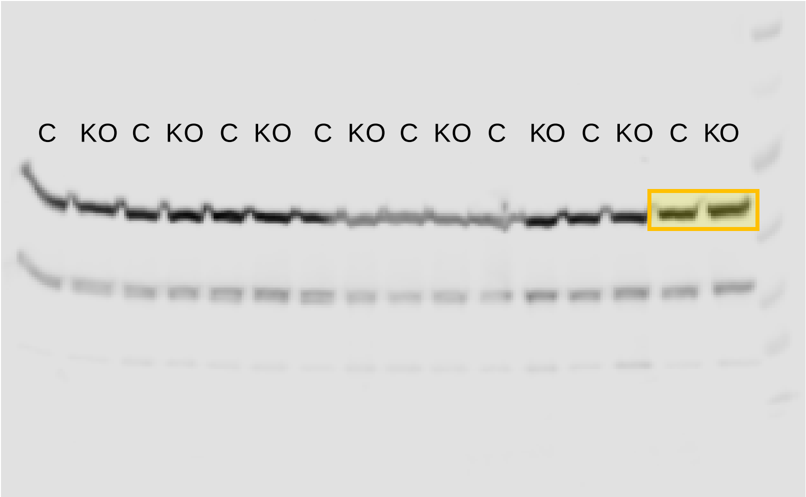

KO = *Depdc5* KO
C = control

Full unedited gel for Figure 2B

pS6

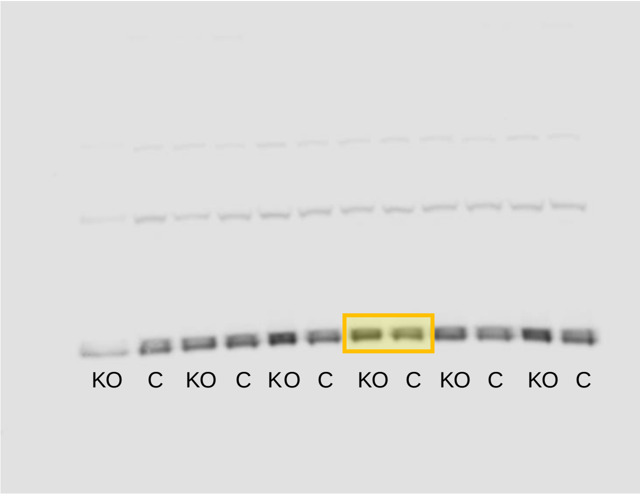

Total S6

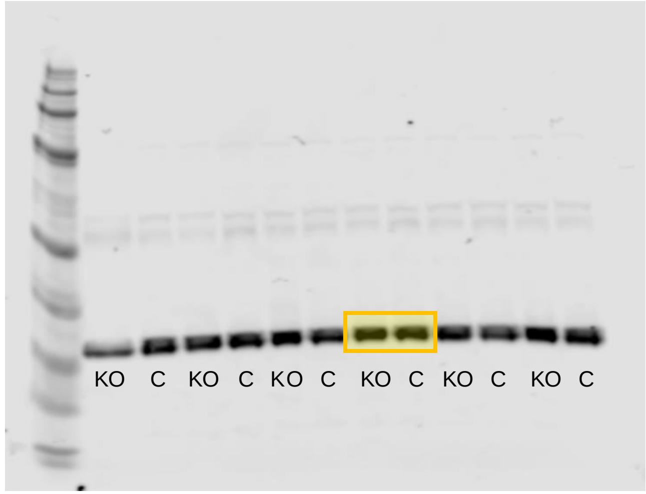

KO = *Depdc5* KO
C = control

pAKT

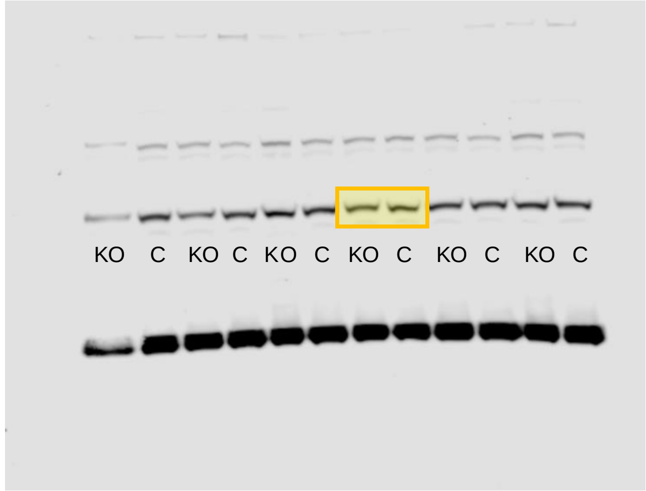

Total AKT

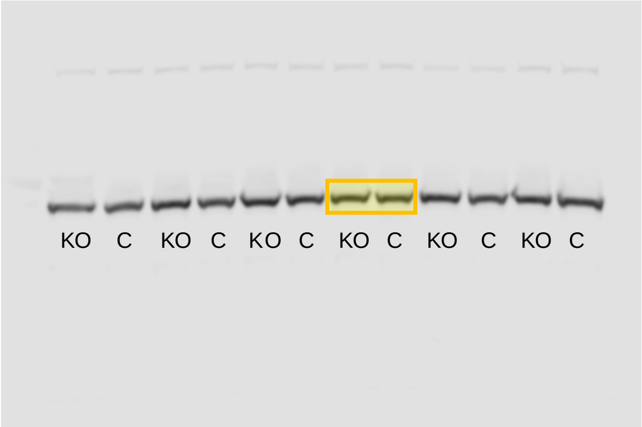

Full unedited gel for Figure 3A

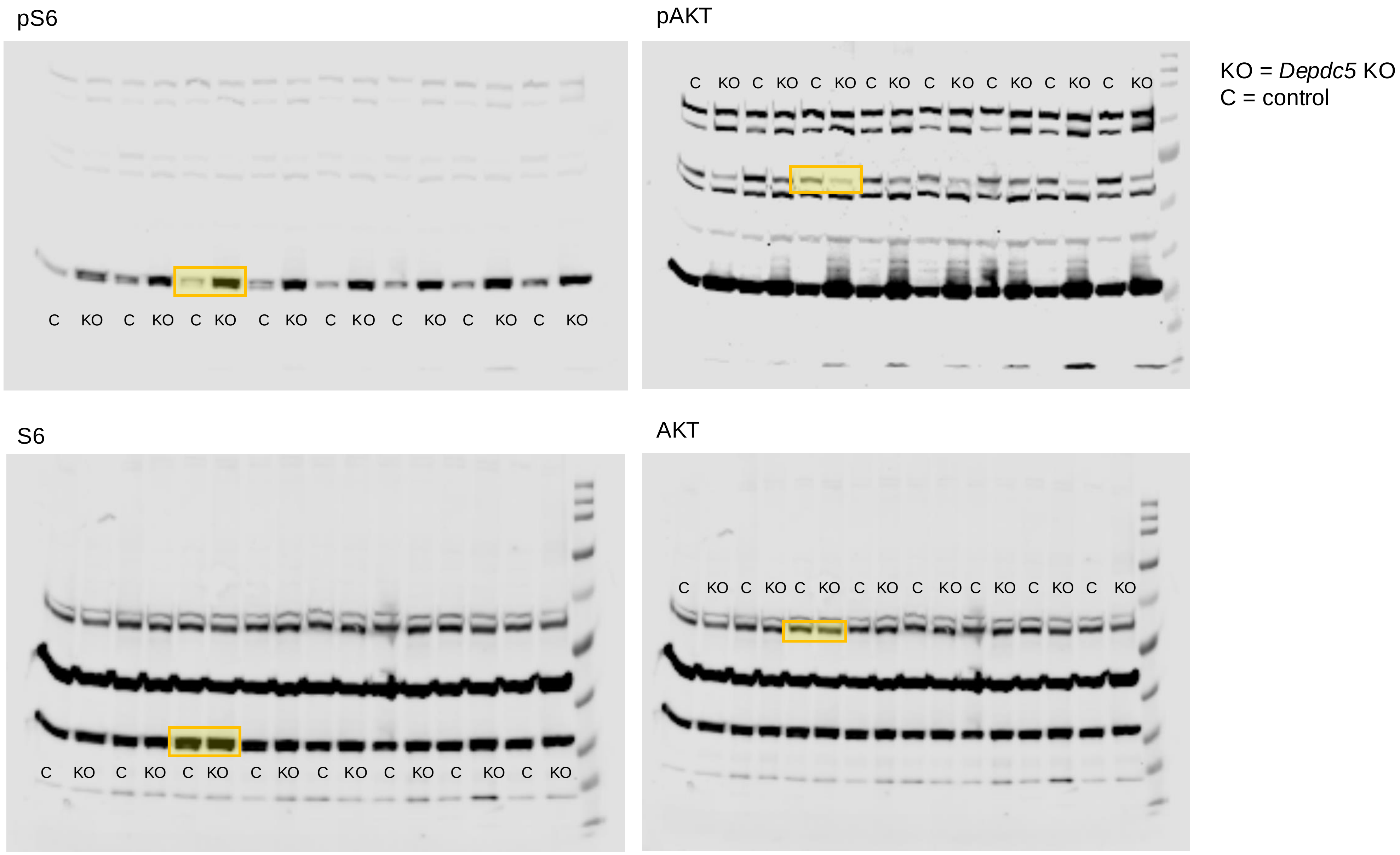

Full unedited gel for Figure 4A

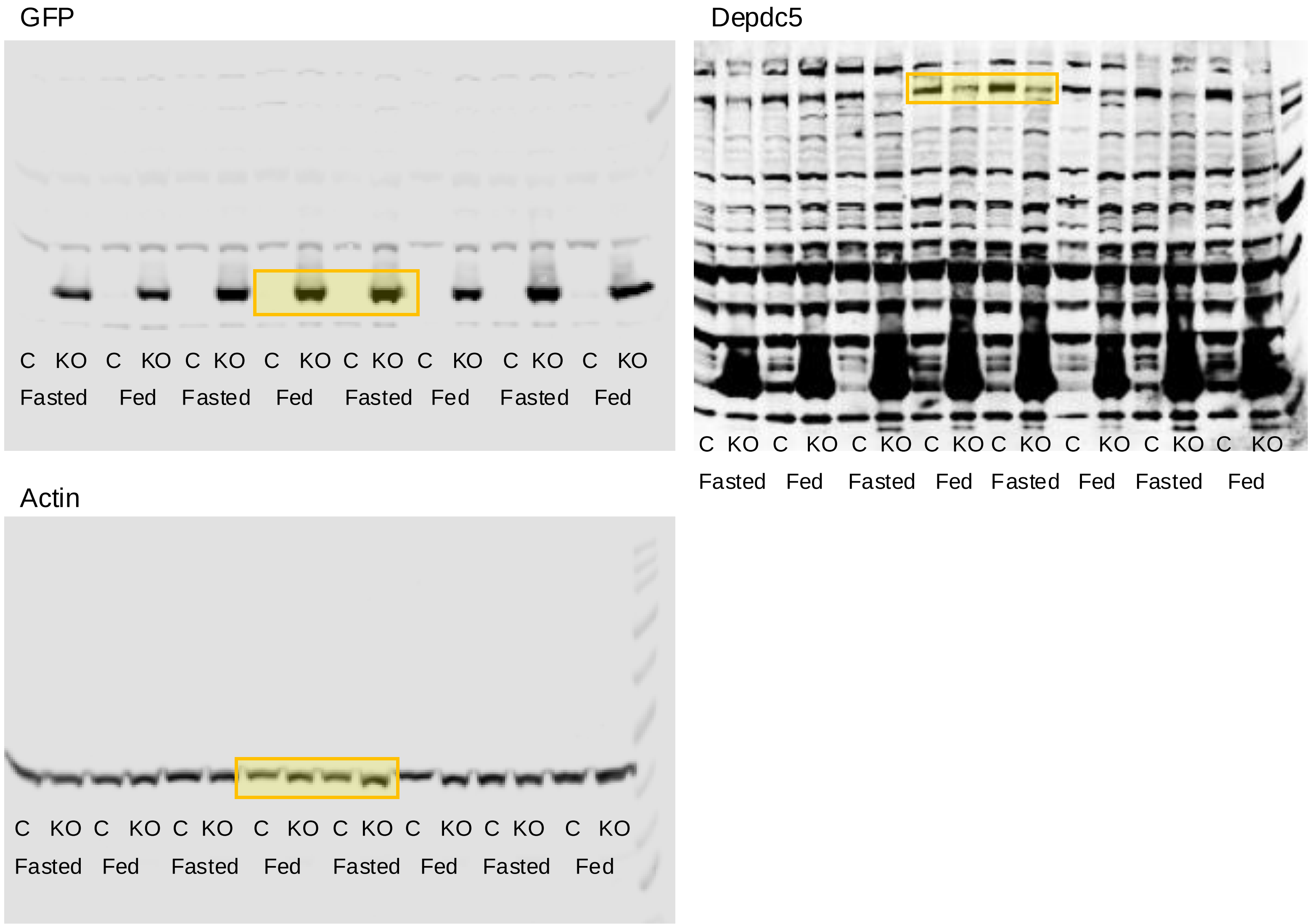

KO = *Depdc5* KO
C = control

Full unedited gel for Figure 7D (1/2)

pS6

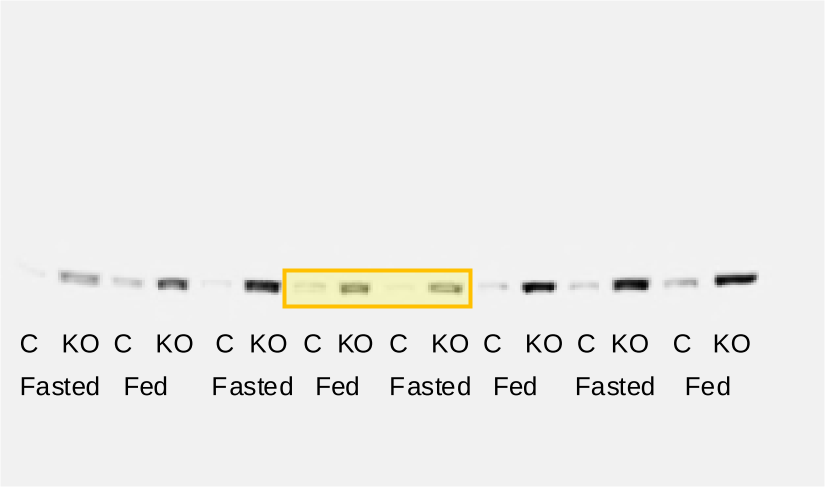

pAKT

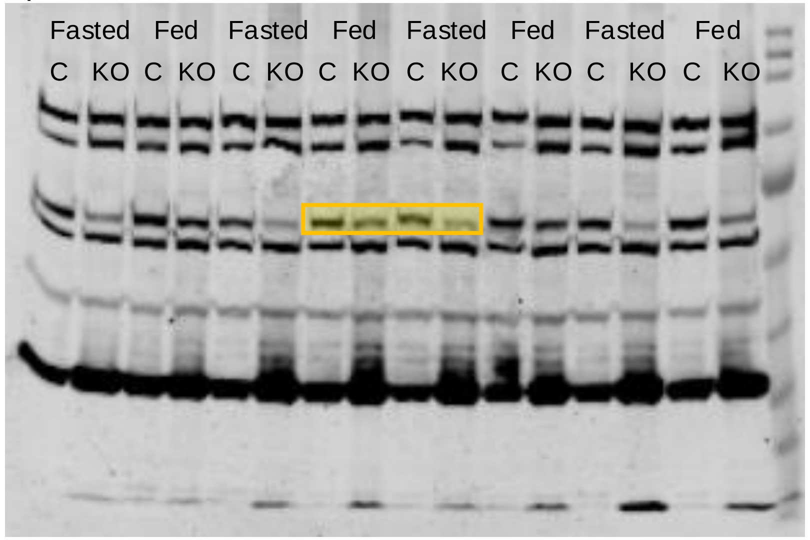

KO = *Depdc5* KO
C = control

Total S6

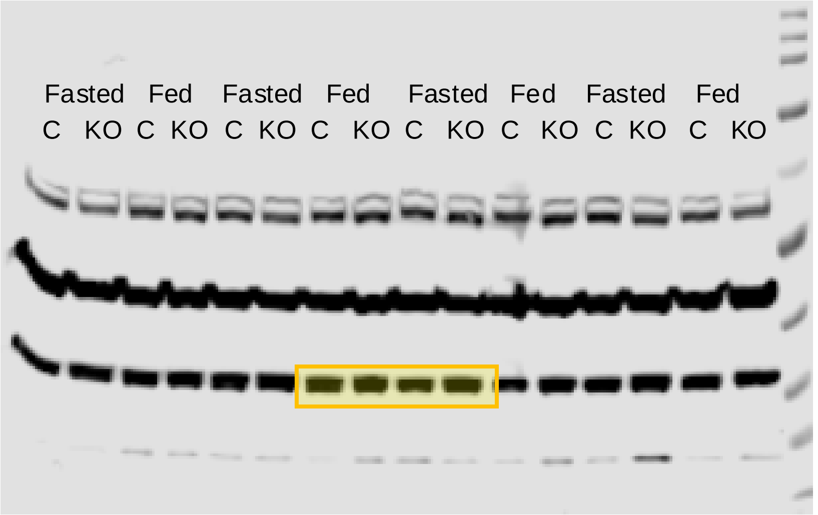

Total AKT

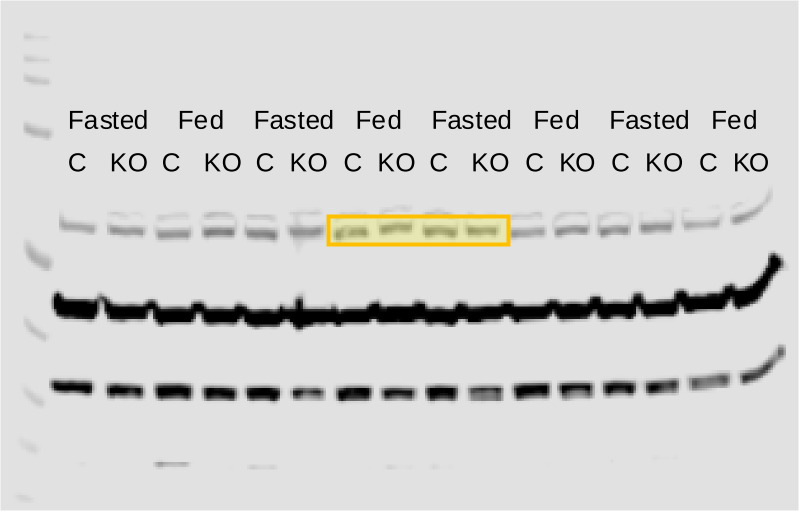

Full unedited gel for Figure 7D (2/2)

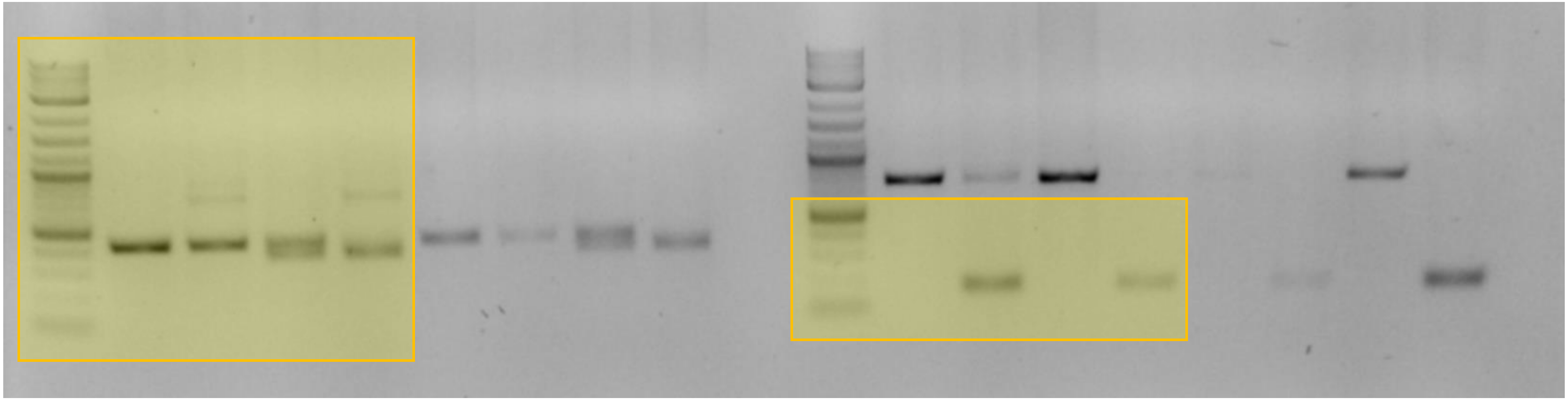

Full unedited gel for Supplemental Figure 4
